# Foliar magnesium enhances rice salt tolerance by improving photosynthesis and regulating ion homeostasis

**DOI:** 10.3389/fpls.2025.1578023

**Published:** 2025-06-16

**Authors:** Yang Liu, Qi Zhu, Rongkai Li, Renyuan Wei, Xiaoyu Geng, Xiang Zhang, Huanhe Wei, Pinglei Gao, Ke Xu, Qigen Dai, Yinglong Chen

**Affiliations:** Jiangsu Key Laboratory of Crop Cultivation and Physiology/Jiangsu Key Laboratory of Crop Genetics and Physiology/Jiangsu Co-Innovation Center for Modern Production Technology of Grain Crops/Key Laboratory of Saline-Alkali Soil Reclamation and Utilization in Coastal Areas, The Ministry of Agriculture and Rural Affairs of China/Research Institute of Rice Industrial Engineering Technology, Yangzhou University, Yangzhou, China

**Keywords:** rice (*Oryza sativa L*.), salinity stress, magnesium, coastal saline-alkali land, ion homeostasis

## Abstract

**Introduction:**

How to increase crop yield in coastal saline-alkali land has become a focus and hot topic of concern for researchers.

**Methods:**

Field experiments were conducted to identify whether foliar application of magnesium sulfate (MgSO_4_·7H_2_O) can enhance rice salt tolerance and improve rice yield. Treatments with four concentrations of MgSO_4_·7H_2_O (10 g L^-1^, 20 g L^-1^, 30 g L^-1^, and 40 g L^-1^) were applied during the jointing and heading stages of rice in three fields with different salt levels in Yancheng City, Jiangsu Province, China in 2022 and 2023.

**Results:**

Results showed that the application of magnesium sulfate, even the lowest concentration of MgSO_4_, could significantly increase the rice yield and total biomass under all the three salt treatments, while the increase displayed more obvious under higher salt treatment. Magnesium sulfate treatment enhanced the Rubisco enzyme activity and total chlorophyll content in rice flag leaves, delayed leaf tip wilt, and thus improved the photosynthetic capacity of rice. Additionally, magnesium sulfate treatment significantly reduced the accumulation of toxic sodium ions (Na^+^) in rice compared to the untreated control, accompanied with notable enhancement of Mg/Na, K/Na, P/Na, and Ca/Na.

**Discussion:**

This study found that magnesium sulfate could enhance the salt tolerance of rice in coastal saline-alkali soils, whereas the effects vary significantly among different concentrations. Under 20 g L^-^¹ of MgSO_4_ treatment, rice leaves exhibited the highest net photosynthetic rate and total chlorophyll content, while the incidence of leaf tip wilt and the accumulation of toxic sodium ions (Na^+^) were minimized, resulting in the highest yield and total biomass. Therefore, 20 g L^-^¹ of MgSO_4_ is likely to be recommended as the optimal application concentration in saline-alkali areas.

## 1 Introduction

Soil salinization is one of the significant abiotic stresses that constrain crop production and yield formation, gradually evolving into a global issue (Zhang et al., 2024; Pu et al., 2014). Approximately 800 million hectares of saline-alkaline land exist worldwide (Fahad et al., 2014). However, this figure continues to rise due to issues such as the use of contaminated water for irrigation and the accumulation of excessive salts resulting from improper irrigation and drainage practices. China is facing increasing scarcity of arable land due to the continuous advancement of urbanization and industrialization, with the per capita arable land area reaching only 40% of the world average (Zhang, 2015). China currently possesses approximately 2.34 million hectares of coastal saline-alkaline land and early 100 million hectares of inland saline-alkaline land, which are considered as potential reserve arable land resources (Li et al., 2022; Geng et al., 2024). Rice (*Oryza sativa L.*) serves as a pioneering crop for the improvement of saline-alkali lands, with its cultivation area on such lands expanding annually (Yan and He, 2010; Wang et al., 2022). It is also one of the most important staple crops globally, expected to be the primary food source for approximately 9 billion people by 2050. To meet this demand, rice production must increase by at least 60%.

Rice is more susceptible to rhizosphere salt stress than wheat, oilseed rape and other crops. Research indicates that while soil salinity exceeds 3‰, the number of effective panicles and biomass in rice are significantly reduced, ultimately leading to a yield decrease of over 30% (Eynard et al., 2005; Chen et al., 2022b). Excessive accumulation of salt ions affects the growth and development of rice by altering its osmotic homeostasis, ionic homeostasis, and oxidative stress. Under low to moderate salt concentrations, salt stress causes stomata closure and reduces the photosynthetic area in rice leaves. Under moderate to high salt concentrations, leaf tip necrosis is frequently occurred (Tester, 2003), mainly due to the excessive accumulation of toxic sodium (Na^+^) in plant tissues, which also disrupts ion balance, competitively inhibits the uptake of essential nutrients such as phosphorus (P), potassium (K), and calcium (Ca), and generates reactive oxygen species (ROS). Excessive reactive oxygen species (ROS) damage genetic material, leading to peroxidation of leaf cell membranes, which in turn produces excessive amounts of malondialdehyde (MDA), hydrogen peroxide (H_2_O_2_), and other peroxides, thereby disrupting the normal growth and development of rice (Tester, 2003; Wu et al., 2022).

Magnesium, as the second most abundant free divalent cation in plant growth processes (Mao et al., 2014), plays a crucial role in numerous vital physiological and biochemical processes such as chlorophyll synthesis and degradation, and the activation of various antioxidant system enzymes (Chen et al., 2018; Tian et al., 2021). Exogenous magnesium supplementation during the growth of plants such as sunflower (Rivelli et al., 2010) and citrus can effectively regulate chlorophyll content and alleviate the normal growth of plants under abiotic stresses like high light conditions. Research has shown that under salt stress, the uptake of magnesium by plants significantly decreases (Yildirim et al., 2008). The application of exogenous magnesium enhances the antioxidant system and modulates gene expression, thus promotes the accumulation of biomass in leaves and buds in castor oil plants (Brito Neto et al., 2012), chili peppers (Sevgin Zirek and Uzal, 2020), and peanut seedlings (Wang et al., 2024). Besides, exogenous magnesium significantly improves the salt tolerance of rice seedlings through the combined effects of enhancing secondary metabolic synthesis pathways and increasing antioxidant enzyme activity (Xuan et al., 2022). Given that previous studies primarily focused on small-scale ecological experiments such as growth chamber or pot trials, this experiment was designed to investigate the effects of foliar supplementation with magnesium sulfate under field conditions in coastal saline-alkali soils. The study aimed to clarify the effect of exogenous magnesium on alleviating salt stress in rice from the perspective of the whole growth period in coastal filed, meanwhile provide the proper concentration of foliar magnesium and clarify the responded mechanism.

## 2 Materials and methods

### 2.1 Materials and treatments

The study was conducted from 2022 to 2023 at the Jinhai Island Base in Sheyang County, Yancheng City, Jiangsu Province (33°57′N, 120°24′E), located less than 5 km from the Yellow Sea. The study area belongs to maritime humid climate with an annual average of approximately 2200 hours of sunshine and an annual rainfall of about 1000 millimeters. The soil type is sandy loam and the pH was 8.0 to 8.5, with 0.46 g kg^−1^ total nitrogen (N), 20.5 mg kg^−1^ olsen phosphorus (P), 85.6 mg kg^−1^ available potassium (K), 55.35 mg g^−1^ total sodium content, 3.04 mg g^−1^ total magnesium content, 24.41 mg g^−1^ total calcium content and 2.7 g kg^−1^organic carbon in the 0–20 cm soil layer. Rice cultivar Nanjing 9108 was adopted and sown on May 12. Rice seedlings with two fully expanded leaves were transplanted to field on June 5. The electrical conductivity of soil in trial field was measured before the experiment, based on which the field was subsequently categorized into three salt treatments, denoted as S1, S2 and S3, respectively, with the increasing of salt content (Chen et al., 2022a). The magnesium sulfate (MgSO_4_·7H_2_O) treatments contained four concentrations of 10 g L^-1^, 20 g L^-1^, 30 g L^-1^ and 40 g L^-1^, denoted as Mg1, Mg2, Mg3, and Mg4, respectively. The magnesium sulfate was foliar sprayed during the jointing (77 days after sowing in 2022 and 78 days in 2023) and heading stages (106 days after sowing in 2022 and 107 days in 2023) of rice with the volume of 380 L ha^-1^, while the control (CK) received an equivalent volume of pure water. Using a hand sprayer, the spray was evenly applied to the rice leaves from 5:00 a.m. to 6:00 a.m. on the day of the experiment, with each experimental plot covering an area of 50 m^2^ and replicated three times. Nitrogen, phosphorus, and potassium were supplied through urea (42% N), superphosphate (12% P_2_O_5_), and potassium chloride (60% K_2_O), respectively. The application rate of pure nitrogen was 262.5 kg ha^-^¹, with a nitrogen fertilizer ratio of 6:4 for basal and tillering fertilizer to panicle fertilizer. The application rate of pure phosphorus was 126 kg ha^-^¹, with a phosphorus fertilizer ratio of 5:5 for basal and tillering fertilizer to panicle fertilizer. The application rate of pure potassium was 360 kg ha^-^¹, with a potassium fertilizer ratio of 4:6 for basal and tillering fertilizer to panicle fertilizer. Other management practices and pest control were conducted according to conventional high-yield cultivation standards.

### 2.2 Sampling and measurement

#### 2.2.1 Soil salinity dynamic monitoring

From 2022 to 2023, during the transplanting to maturity phase, soil conductivity in each plot was measured every 5 days using the Spectrum ECtestr-2265FS (Spectrum, USA).

#### 2.2.2 Rice grain yield and its components

After the rice maturation, 100 complete plants were selected from each treatment for yield determination, with two replications. The number of panicles per experimental plot, the number of grains per panicle, and the thousand-grain weight were measured, and the filled-grain percentage was calculated.

Thousand-grain weight was calculated by randomly selecting 1000 plump seeds per replicate and averaging their weights.

Filled-grain percentage = (number of filled grains per panicle)/(total number of grains per panicle).

#### 2.2.3 Dry matter weight determination

At the jointing (77 days after sowing in 2022 and 78 days in 2023), heading (106 days after sowing in 2022 and 107 days in 2023), and maturity stages (153 days after sowing in 2022 and 158 days in 2023), 5 holes of rice plants were sampled based on the average number of tillers per plot to determine the dry matter weight. The sampled plants were divided into leaves, stems, and panicles, then inactivation at 105°C for 30 minutes and dried at 80°C for 3 days until constant weight. Crop growth rate (g m^-2^ d^-1^) = (W2 - W1)/(t2 - t1), where W1 and W2 represent the dry matter weights of the population measured during two distinct periods, and t1 and t2 denote the time (days) corresponding to the two population dry matter weight measurements.

#### 2.2.4 Photosynthetic parameters and SPAD values

At 20 days post-heading, the photosynthetic parameters including net photosynthetic rate, transpiration rate, stomatal conductance, and intercellular CO_2_ concentration of the flag leaves were measured using a portable photosynthesis system (LI-6400, LI-COR, Lincoln, NE, USA). The settings were based on Gu (Gu et al., 2017) until stable photosynthetic data were obtained, after which the leaves were removed from the chamber.

SPAD values were measured at the base, middle, and tip of the uppermost fully expanded leaf at the jointing, heading, and grain-filling stages using a SPAD-502 chlorophyll meter (MINOLTA, Japan), with six uniform rice plants selected per treatment.

#### 2.2.5 Leaf physiological measurements

At 20 days post-heading, the flag leaves of each treatment were collected between 9:00-10:00 AM, with 15–20 leaves collected per treatment. After collection, the leaves were trimmed of their tips, tails, and veins, and immediately immersed in liquid nitrogen for freezing. Once fully frozen, the samples were transferred to a -80°C freezer for storage, to be used for subsequent analytical measurements.

#### 2.2.6 Physiological parameter measurements

Leaf tip wilt index was determined of all the rice leaves at the jointing, heading, 15, 30, and 45 days after heading stage using the formula TWI = L1/L2, where L1 is the length of the wilted part of the all leaves and L2 is the total length of all leaves (Chen et al., 2021).

Superoxide dismutase (SOD) activity was measured using the NBT photochemical reduction method (Jin et al., 2008); peroxidase (POD) activity was measured using the guaiacol method (Jiang et al., 2021); catalase (CAT) activity was measured using UV spectrophotometry (Panda et al., 2017); ascorbate peroxidase (APX) activity was measured using UV spectrophotometry (Molina et al., 2002); malondialdehyde (MDA) was measured according to Di (Di et al., 2021); soluble sugar was measured according to Wang (Wang et al., 2014); soluble protein was measured according to Eva M et al (Campion et al., 2011); free proline content was measured according to Vieira (Vieira et al., 2010).

Leaf Rubisco enzyme activity, H^+^-ATPase enzyme activity, and hydrogen peroxide (H_2_O_2_) content were determined using reagent kits from Keming Biotechnology Co., Ltd. (Suzhou).

#### 2.2.7 Element content in organs

At the jointing, heading, and maturity stages, three holes of plants were sampled, divided into stems, leaves, and panicles, inactivation at 105°C for 30 minutes, and dried at 80°C until constant weight. The dried samples were then ground and sieved. The contents of Na, Mg, K, P, and Ca in the samples were measured using an inductively coupled plasma mass spectrometer (AGILENT 7900 ICP-MS, Agilent Technologies, Japan), with three replicates per treatment.

### 2.3 Data statistics and analysis

Multivariate analyses of variance were carried out to determine the effects of different salinity concentrations and magnesium sulfate treatments, as well as their interactions on rice grain yield and its components, leaf tip wilt index, ion content, and distribution at a significance level of 5%. Additionally, pairwise comparisons were also performed on treatment means by using the LSD test at a significance level of 5%. The clustering is ward’s method and the clustering distance is Euclidean. All analyses were performed using IBM SPSS Statistics 26 (IBM, Armonk, NY, USA). Graphs appearing in articles such as polarized heat maps and Pearson correlation plots were prepared using Origin 2024b (Origin Lab, Hampton, MA, USA).

## 3 Results

### 3.1 Dynamic changes of soil electrical conductivity in different saline-alkaline soils

**Figure 1:**
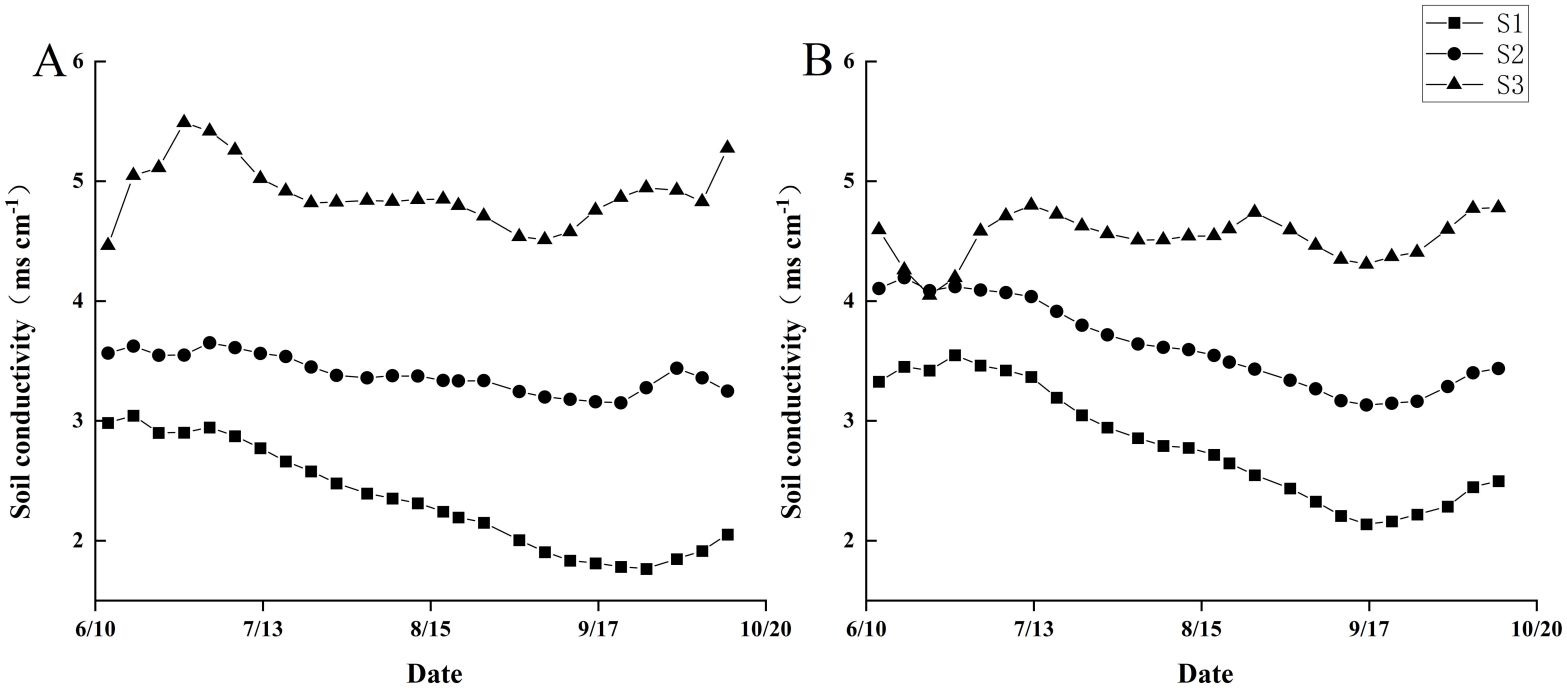
Dynamic changes of soil electrical conductivity in the three trial fields during rice growth season from 2022 to 2023. **(A)** shows the year 2022 and **(B)** shows the year 2023.

As shown in **Figure 1**, the analysis reveals that during the 2022–2023 trials, the trends in soil electrical conductivity (EC) after rice transplanting were consistent across the three different saline-alkaline fields. Over the two-year period, the average electrical conductivity in three fields was 2.54 mS cm^-1^ (S1), 3.48 mS cm^-1^ (S2) and 4.7 mS cm^-1^ (S3), respectively.

### 3.2 Rice grain yield and its components, dry matter weight and harvest index

As shown in **Table 1**, salinity significantly decreased rice yield and the decrease displayed more obvious with the increasing of salinity level. Compared to mild saline-alkali soil (S1), average yield reduction under severe saline-alkali soil (S3) reached 33%, with an average decline of 18.6% in filled-grain percentage and 7.6% in 1000-grain weight due to the elevated salinity. Overall, the decline in rice yield due to salinity was notably alleviated after foliar applying magnesium sulfate in all the three saline fields. The effects of Mg1 to Mg4 treatments from 2022 to 2023 exhibited a trend of first increasing and then decreasing. However, significant differences were observed in the effects of different magnesium sulfate concentrations. Compared to that of control, rice yield increased the most under spraying concentration of 20 g L^-1^ MgSO_4_ under all the three salt levels. The rice yield was increased averagely by 15%, 37.9% and 47.2% under S1, S2 and S3 salt concentration, respectively, across the two years. In terms of the number of panicles, significant difference occurred among different salt concentrations under the same magnesium sulfate concentration, where the number of panicles decreased with increased salt concentration and the most significant difference appeared under the S1 treatment. Under the same salt concentration, the Mg2 treatment was the most effective among different magnesium sulfate concentrations, followed by Mg1 and Mg3, while Mg4 was the least effective and showed no significant difference from the control (CK). At S1 salt concentration, the two-year average increase was 5.5% for Mg1 treatment, 4% for Mg2 treatment, 3.9% for Mg3 treatment, and 2.7% for Mg4 treatment. At S2 salt concentration, the average increase for the treatments was 18.6% (Mg1), 18.3% (Mg2), 17.7% (Mg3), and 12.4% (Mg4), whereas at S3 treatment, the average increase amounted to 14.4% for Mg1 treatment, 8.2% for Mg2 treatment, 13.3% for Mg3 treatment and 4% for Mg4 treatment. The effects of different magnesium sulfate concentrations varied in terms of panicle grain number over the two years, but overall, Mg2 and Mg3 treatments were the most effective, with Mg1 and Mg4 being less so. The Mg2 treatment significantly improved the filled-grain percentage by 10.2% (S1), 17.2% (S2), 24.2% (S3) in 2022, and 4.4% (S1), 11.1% (S2), 23.5% (S3) in 2023, respectively, compared to CK. Across the three salt concentrations, the average filled-grain percentage under Mg1, Mg2, Mg3 and Mg4 were 88%, 91.2%, 83.7% and 83.4%, in 2022, and 78.2%, 85.2%, 73.1% and 73.4%, respectively. The effect of magnesium sulfate treatment on the 1000-grain weight varied at different salt concentrations, with Mg1 being the most effective, increasing by up to 2.9% (2022 S1), followed by Mg2, Mg3, and Mg4. However, there was an overall upward trend. As shown in **Table 2**, the analysis reveals that across both years of the experiment, the dry matter weight and growth rate of the rice population at various stages significantly decreased with increasing salt concentration. Compared to the S1 and S2, the dry matter weight at maturity decreased more significantly under S3, with values of 10.6 t ha^-1^ and 9.6 t ha^-1^ in 2022 and 2023, respectively. Magnesium sulfate treatments at different concentrations significantly enhanced the dry matter weight of the rice population at all stages, with significant differences among treatments. Overall, the Mg2 treatment proved to be the most effective. Compared to the control treatment, the Mg2 treatment in 2022 increased the dry matter weight at maturity by 21.4% (S1), 48.4% (S2), and 41.2% (S3), and in 2023 by 12% (S1), 9.5% (S2), and 48.8% (S3). In the 2022–2023 experiments, the harvest index showed a trend of first decreasing and then increasing with rising salt concentrations. There was a significant interaction effect between magnesium sulfate treatments at different concentrations and different salt concentration treatments on the dry matter weight, crop growth rate, and harvest index of the rice population.

**Table 1:** Effects of magnesium application on rice yield and its components.

Treatment		Number of panicles (×10^4^ ha^-1^)		Spikelets per panicle		Filled-grain percentage (%)		1000-grain weight (g)		Yield (t ha^-1^)	
		2022	2023	2022	2023	2022	2023	2022	2023	2022	2023
S1	CK	319.1Aa	252.5Aa	103.3ABa	156.8Ba	86.2Ca	82.4Aa	26.8Ba	23.1Ca	7.6CDa	6.6Ca
	Mg1	319.1Aa	280.3Aa	102.5Bab	147.0Cb	92.3Ba	82.4Aa	27.5Aa	24.1Ba	8.0Ba	7.5Ba
	Mg2	333.0Aa	261.9Aa	108.1Aa	154.0Bc	95.0Aa	86.0Aa	25.5Ca	24.6Aa	8.6Aa	7.8Aa
	Mg3	333.0Aa	261.2Aa	101.8Bb	166.0Ab	90.7Ba	69.9Ba	25.9Ca	24.2Ba	7.8BCa	6.8Cb
	Mg4	319.1Aa	266.1Aa	106.6ABab	156.0Bb	92.2Ba	73.1Ba	24.2Da	23.9Ba	7.4Da	6.7Ca
S2	CK	277.5Bab	190.1Aa	111.6Aa	153.4Ba	79.1Cb	77.0Bb	24.1Bb	22.8Ba	5.9Db	5.1Db
	Mg1	305.3ABa	241.8Aa	107.0Ba	167.2Aab	86.0Bb	76.2Ca	25.9Ab	23.6Aa	7.1Bb	6.5BCb
	Mg2	319.1Aa	231.2Aab	104.3Ba	175.0Ab	92.7Aa	85.5Aa	25.6Aa	22.9Bb	7.7Ab	7.4Aab
	Mg3	305.3ABa	238.3Aab	113.6Aa	175.9Aa	82.6BCb	71.2Da	25.4Ab	23.2Bb	7.3Bb	7.0ABa
	Mg4	291.4ABa	227.9Ab	114.2Aa	172.5Aa	81.0Cb	70.3Da	23.7Ba	23.6Aa	6.4Cb	6.2Ca
S3	CK	263.6Cb	189.0Aa	108.9Aa	166.1Ca	69.1Cc	68.0Cc	24.5Ab	21.6Bb	5.0Cc	4.5Db
	Mg1	305.3ABa	213.7Aa	98.0Bb	176.3Ba	85.7Ab	76.0Ba	25.5Ab	22.6Ab	6.4Bc	6.2Bb
	Mg2	291.4BCa	200.1Ab	107.7Aa	186.8Aa	85.8Aa	84.0Aa	25.5Aa	22.7Ab	6.8Ac	7.0Ab
	Mg3	305.3Aa	209.5Ab	98.7Bb	180.2ABa	77.7Bc	78.0ABa	25.1Ab	22.1Bc	6.1Bc	6.2BCc
	Mg4	277.5BCa	194.3Ac	97.6Bb	179.2ABa	77.0Bc	76.7Ba	24.3Aa	21.8Bb	5.1Cc	5.7Ca
F value	S	*		*		*		*		*	
	Mg	*		*		*		*		*	
	S×Mg	ns		*		*		*		*	

S1, S2 and S3 represent mild, medium and severe saline-alkali soil, respectively. Mg1, Mg2 Mg3 and Mg4 represent 10, 20, 30 and 40 g L^-1^magnesium application, respectively. Within the same saline-alkaline soil, the values with different uppercase letters indicate significant differences in different magnesium application at a significance level of 5%; within the same magnesium application, the values with different lowercase letters indicate significant differences in different saline-alkaline soil at a significance level of 5%. “*” and “ns” indicate significance and no significance, respectively, according to the LSD test at a 5% level of probability.

**Table 2:** Effects of magnesium application dry matter weight and harvest index of rice.

Treatment		Dry matter weight of population (t ha^-1^)						Crop growth rate (g m^-2^ d^-1^)				Harvest index	
		Jointing Stage		Heading Stage		Maturity Stage		Jointing-Heading		Heading-Maturity			
		2022	2023	2022	2023	2022	2023	2022	2023	2022	2023	2022	2023
S1	CK	4.5Ba	4.5Ca	11.3Cb	11.5Ba	15.6Ba	18.3Ba	22.6Bb	24.2Bab	9.1Aa	10.4Aa	0.487ABa	0.362Bb
	Mg1	4.8Aa	4.5Ca	12.6Bb	11.4Ba	15.5Bb	19.1Ba	26.1Bb	23.9Bb	6.2Ba	11.8Aa	0.517Aa	0.392Aa
	Mg2	4.9Aa	4.8Ba	14.0Ab	13.0Aa	18.9Aa	20.4Aa	30.4Ab	28.5Aa	10.5Aa	11.4Aa	0.452Ba	0.380ABc
	Mg3	4.7Aa	5.0Aa	12.4BCb	12.1Ba	14.8Bb	18.8Ba	25.5Bb	24.4Ba	5.1Bb	10.3Aab	0.524Aa	0.364ABb
	Mg4	4.9Aa	5.0Aa	12.0BCa	11.9Ba	14.8Ba	18.4Ba	23.8Bb	23.8Ba	5.8Ba	10.0Aa	0.499Aa	0.367ABb
S2	CK	3.5Bb	3.5Ab	12.4Da	11.2ABa	13.8Db	16.7ABb	29.8Da	26.4Aa	2.8Db	8.4Cb	0.429Ab	0.307Bc
	Mg1	3.9ABb	3.6Ab	15.0Ba	11.2ABb	18.4Ba	16.0ABab	36.8Ba	26.2Aa	7.2BCa	7.3Cab	0.386Bc	0.405Aa
	Mg2	4.0Ab	3.4ABc	15.7Aa	11.3Ab	20.4Aa	18.2Aa	38.9Aa	27.3Aab	10.1ABa	10.7Bab	0.379Bb	0.407Ab
	Mg3	3.7ABb	3.0BCb	13.6Ca	10.5BCab	19.6ABa	18.4BCa	33.0ABa	25.9Aa	12.8Aa	12.2Aa	0.372Bb	0.379Ab
	Mg4	3.9ABb	2.9Cb	13.4Ca	9.9Cb	15.7Ca	16.9Ca	31.6Ca	24.0Aa	4.8CDa	10.8ABa	0.406ABa	0.367ABb
S3	CK	3.0Bc	3.1Cc	9.2Dc	8.8Bb	10.6Dc	9.6Bc	20.9Db	19.6Bb	2.9Bb	1.4Cc	0.470Aab	0.465BCa
	Mg1	3.3Ac	3.4Bb	10.9Bc	10.4Ac	13.3Bc	14.1Ab	25.1Bb	23.9Ab	5.3ABa	5.8ABb	0.478Ab	0.438Ca
	Mg2	3.4Ac	3.7Ab	12.6Ac	10.6Ab	15.0Ab	14.4Ab	30.7Ab	23.8Ab	5.1ABa	5.7ABb	0.457Aa	0.490ABa
	Mg3	3.3Ab	3.1Cb	10.0Cc	9.3Bb	13.0Bb	13.3Bb	22.4Bc	21.4Ba	6.4Ab	6.1Ab	0.472Aa	0.465BCa
	Mg4	3.2ABc	3.3Bb	10.0Cb	9.0Bc	11.6Cb	10.8Bb	22.9Cb	19.8Bb	3.4Ba	2.7BCb	0.439Aa	0.530Aa
F value	S	*		*		*		*		*		*	
	Mg	*		*		*		*		*		ns	
	S×Mg	*		*		*		ns		*		*	

S1, S2 and S3 represent mild, medium and severe saline-alkali soil, respectively. Mg1, Mg2 Mg3 and Mg4 represent 10, 20, 30 and 40 g L^-1^magnesium application, respectively. Within the same saline-alkaline soil, the values with different uppercase letters indicate significant differences in different magnesium application at a significance level of 5%; Within the same magnesium application, the values with different lowercase letters indicate significant differences in different saline-alkaline soil at a significance level of 5%. “*” and “ns” indicate significance and no significance, respectively, according to the LSD test at a 5% level of probability.

### 3.3 Effects of magnesium application on rice leaf tip wilt index

**Figure 2:**
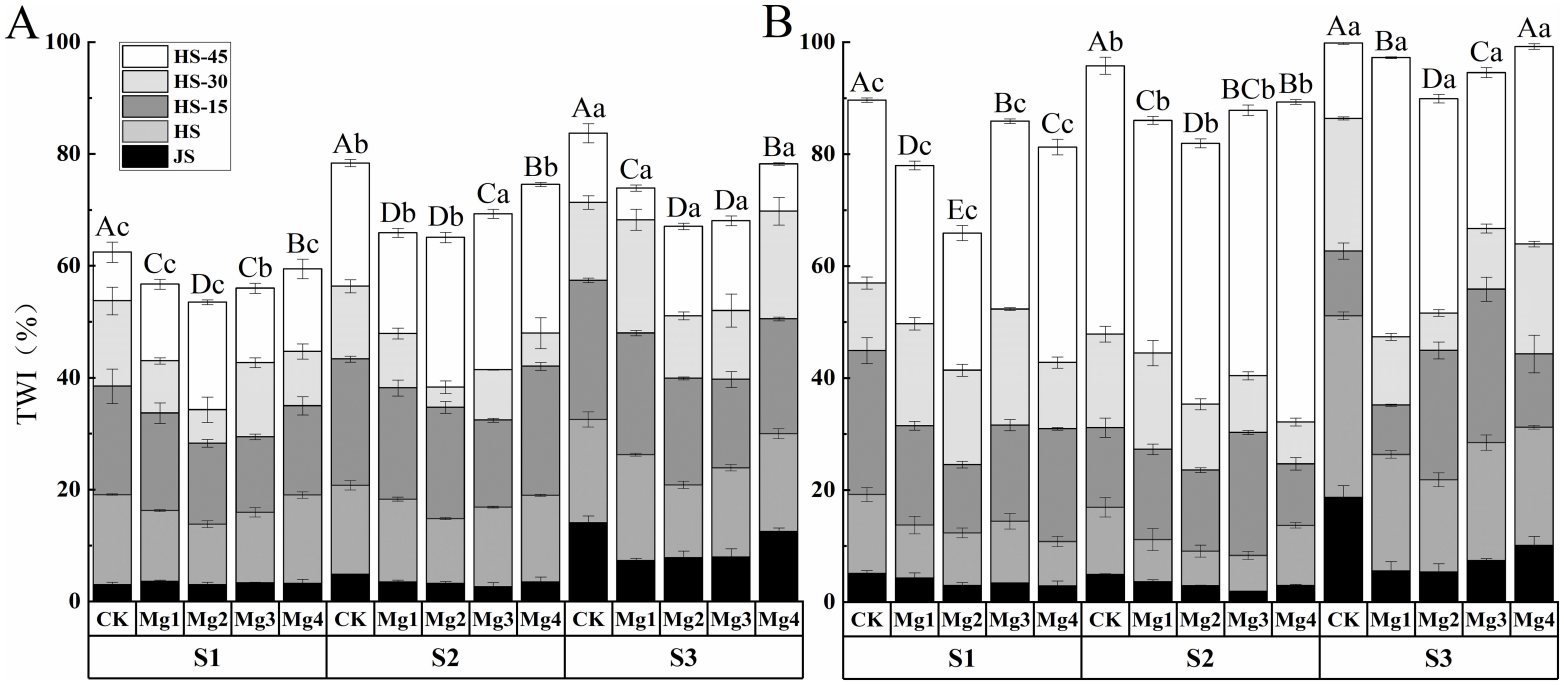
Effects of magnesium application on the rice leaf tip wilt index. **(A)** shows the year 2022 and **(B)** shows the year 2023. JS and HS represent jointing stage and heading stage, respectively. HS-15, HS-30 and HS-45 represent 15, 30 and 45 days, respectively, after the heading stage. S1, S2 and S3 represent mild, medium and severe saline-alkali soil, respectively. Mg1, Mg2 Mg3 and Mg4 represent 10, 20, 30 and 40 g L^-1^ magnesium, respectively. Within the same saline-alkaline soil, the values with different uppercase letters indicate significant differences in different magnesium application at a significance level of 5%; Within the same magnesium application, the values with different lowercase letters indicate significant differences in different saline-alkaline soil at a significance level of 5%.

The dynamic changes in rice leaf tip necrosis under different concentrations of magnesium sulfate application are shown in **Figure 2** for two years of experimentation. The trends of leaf tip necrosis in rice across both years were generally consistent. As the growth process advanced, the condition of leaf tip necrosis gradually worsened. The growth rate was slow before the heading stage, but it accelerated significantly from the heading stage to 45 days post-heading, with the largest increase occurring between 30- and 45- days post-heading. Analysis of the two-year experimental data showed that under different salt concentrations, magnesium sulfate at various concentrations significantly alleviated rice leaf tip necrosis. Compared to the control (CK), the reduction rate was the most significant under Mg2 treatment, which were 14.3% (S1), 17% (S2), and 19.9% (S3) in 2022, and 26.5% (S1), 14.5% (S2), and 10% (S3) in 2023, respectively. At the same salt concentration, there were significant differences among treatments with different concentrations of magnesium sulfate, generally showing a pattern of Mg2 > Mg1 > Mg3 > Mg4. The best treatment effect was observed with Mg2 in both years, with the maximum alleviation of 19.9% in 2022(S3) and 26.5% in 2023(S1) compared to the control.

### 3.4 Effects of magnesium application on rice SPAD values

**Figure 3:**
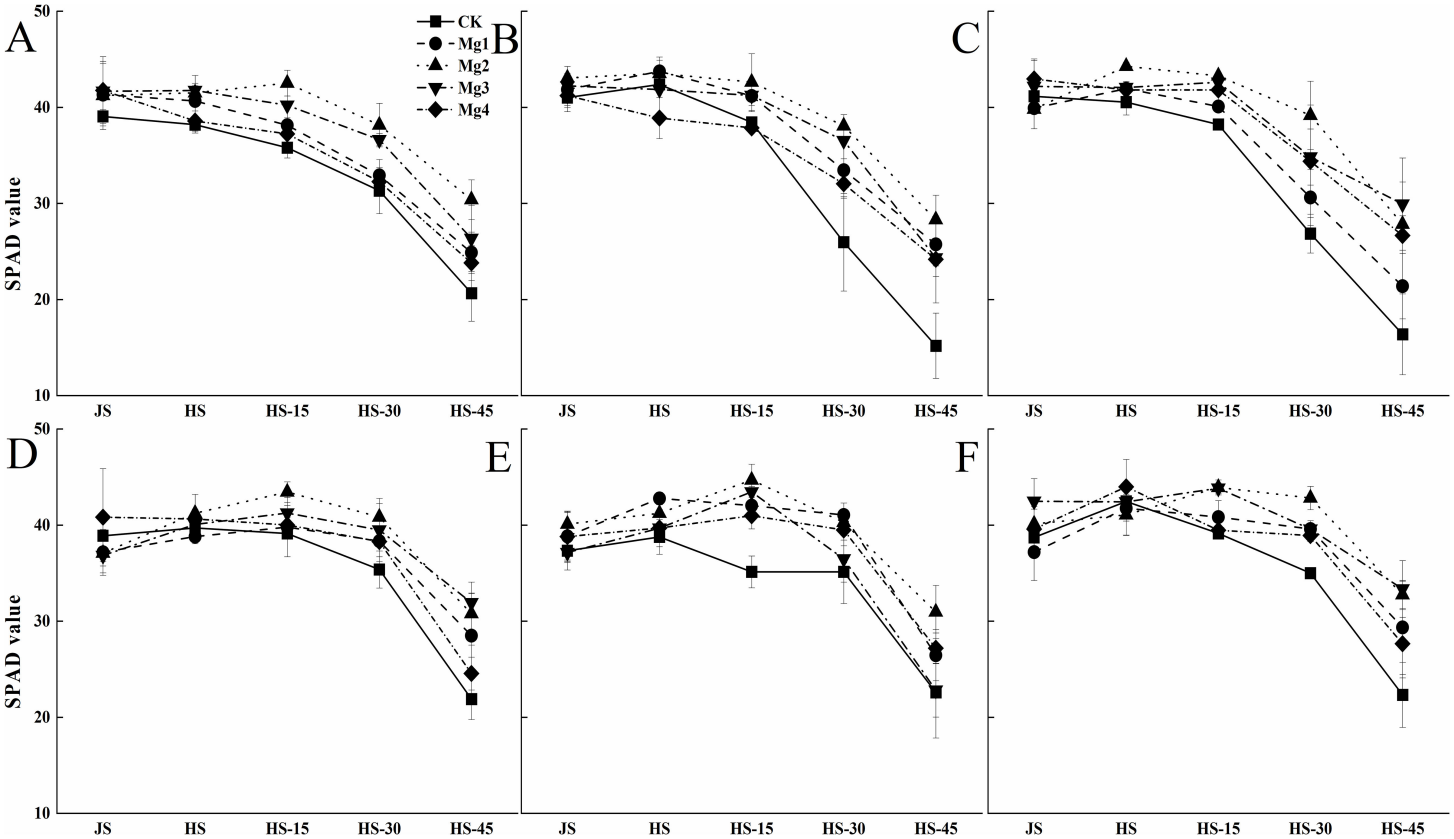
Effects of magnesium application on rice SPAD values. JS and HS represent jointing stage and heading stage, respectively. HS-15, HS-30 and HS-45 represent 15, 30 and 45 days, respectively, after the heading stage. **(A–C)** represent mild, medium and severe saline-alkali soil of 2022, **(D–F)** represent mild, medium and severe saline-alkali soil of 2023, respectively. Mg1, Mg2 Mg3 and Mg4 represent 10, 20, 30 and 40 g L^-1^ magnesium, respectively.

**Figure 3** illustrates the trends in SPAD values of rice at various growth stages under different salt concentrations over two years. It is observed that the SPAD values of rice leaves generally followed a consistent pattern across the years, showing a gradual decline as the growth stages progressed. The SPAD values of the fully expanded last leaf tended to decrease over time. Under the same salt concentration, the peak SPAD values of rice leaves varied after treatments with different concentrations of magnesium sulfate. In 2022, except for the Mg2 treatment, which reached its maximum 15 days after heading, all other magnesium sulfate treatments showed peak SPAD values similar to the control at the heading stage. In 2023, both Mg2 and Mg3 treatments reached their maximum 15 days after heading, while the other treatments exhibited patterns consistent with those observed in 2022. Under different salt concentrations, the effects of magnesium sulfate application varied, with the overall performance ranking as Mg2 > Mg3 > Mg1 > Mg4.

### 3.5 Effects of magnesium application on the pigment composition of rice flag leaf at 20 days after heading

As shown in **Table 3**, the application of magnesium sulfate at different concentrations significantly increased the contents of chlorophyll a, chlorophyll b, and carotenoids in the flag leaves of rice under various salt concentrations, with the enhancement effect ranking as Mg2 > Mg1 > Mg3 > Mg4 > CK. In 2022, the interaction between different salt concentrations and magnesium sulfate application significantly affected the contents of chlorophyll a, chlorophyll b, and total chlorophyll in the flag leaves of rice. In 2023, a significant interaction was observed only for the increase in chlorophyll a content. Over the two years of testing, there was no significant interaction between salt treatment and magnesium sulfate application on the increase in carotenoid content. In both 2022 and 2023, the M2 treatment yielded the best results, with the largest increase. Compared to the control, Mg2 treatment significantly enhanced chlorophyll content in rice flag leaves across two consecutive growing seasons. Chlorophyll a content increased by 58.2% (S1), 34% (S2), and 59.8% (S3) in 2022, with further improvements of 64.3% (S1), 42.8% (S2), and 31.2% (S3) in 2023. Chlorophyll b content exhibited increases of 84.9% (S1), 62.5% (S2), and 78.7% (S3) in 2022, followed by 46.6% (S1), 17.6% (S2), and 36.4% (S3) in 2023. Total chlorophyll (a + b) content showed improvements of 63.6% (S1), 39% (S2), and 63.2% (S3) in 2022, and 59.5% (S1), 35.5% (S2), and 32.6% (S3) in 2023, while carotenoid content was elevated by 70.4% (S1), 30.6% (S2), and 40.9% (S3) in 2022, and 34.5% (S1), 29.3% (S2), and 21.7% (S3) in 2023.

**Table 3:** Effects of magnesium application on the pigment composition of rice flag leaf at 20 days after heading.

Treatment		Chlorophyll a (mg g^-1^ FW)		Chlorophyll b (mg g^-1^ FW)		Chlorophyll a+b (mg g^-1^ FW)		Chlorophyll a/b		Carotenoid (mg g^-1^ FW)	
		2022	2023	2022	2023	2022	2023	2022	2023	2022	2023
S1	CK	0.91Ba	0.82Ca	0.20Ba	0.31Ba	1.10Ba	1.13Ca	5.51Aa	2.67Aa	0.22Dc	0.16Aa
	Mg1	1.15ABb	1.06BCb	0.27ABb	0.39ABb	1.42ABb	1.46ABCb	5.02ABab	2.70Aa	0.32BCb	0.20Aa
	Mg2	1.44Aa	1.35Aa	0.37Aa	0.45Aa	1.81Aa	1.80Aab	4.38Ba	3.03Aa	0.38Aa	0.22Aa
	Mg3	1.13ABa	1.19ABa	0.24ABa	0.41ABa	1.38ABa	1.60ABa	5.33Aa	2.91Aa	0.32Ba	0.19Aa
	Mg4	0.96Ba	0.98BCb	0.21Ba	0.37ABa	1.16Ba	1.35BCa	5.81Aa	2.68Aa	0.28Ca	0.19Aa
S2	CK	0.94Ca	0.93Ca	0.19Cb	0.37Aa	1.12Ca	1.31Ca	6.78Aa	2.49Ab	0.25Cb	0.18Aa
	Mg1	1.19ABb	1.05Bb	0.27Bb	0.40Ab	1.46ABb	1.45BCb	5.31Ba	2.62Aa	0.30Cb	0.21Aa
	Mg2	1.26Aa	1.33Aa	0.30Aa	0.44Aa	1.56Aa	1.77Ab	4.71Ba	3.07Aa	0.33Ab	0.23Aa
	Mg3	1.12ABa	1.08Ba	0.27ABa	0.40Aa	1.39ABa	1.48Ba	4.91Ba	2.67Aa	0.31Aa	0.21Aa
	Mg4	1.10BCa	1.06Bab	0.24Ba	0.39Aa	1.34BCa	1.44BCa	5.27Ba	2.79Aa	0.27Ba	0.20Aa
S3	CK	0.97Ba	1.03Ca	0.21Ca	0.38Ca	1.18Ba	1.41Ca	5.47Aa	2.71Aa	0.30Ba	0.20Aa
	Mg1	1.53Aa	1.33Aa	0.37Aa	0.48ABa	1.90Aa	1.82Aa	4.46Bb	2.77Aa	0.42Aa	0.23Aa
	Mg2	1.55Aa	1.35Aa	0.38ABa	0.52Aa	1.92Aa	1.87Aa	4.56Ba	2.61Aa	0.42Aa	0.24Aa
	Mg3	1.12Ba	1.16Ba	0.25BCa	0.42BCa	1.37Ba	1.58Ba	5.14ABa	2.76Aa	0.34Ba	0.22Aa
	Mg4	1.07Ba	1.09BCa	0.24Ca	0.41Ca	1.31Ba	1.50BCa	5.37Aa	2.69Aa	0.32Ba	0.21Aa
F value	S	*		*		*		ns		*	
	Mg	*		*		*		*		*	
	S×Mg	*		ns		*		ns		ns	

S1, S2 and S3 represent mild, medium and severe saline-alkali soil, respectively. Mg1, Mg2 Mg3 and Mg4 represent 10, 20, 30 and 40 g L^-1^magnesium application, respectively. Within the same saline-alkaline soil, the values with different uppercase letters indicate significant differences in different magnesium application at a significance level of 5%; Within the same magnesium application, the values with different lowercase letters indicate significant differences in different saline-alkaline soil at a significance level of 5%. “*” and “ns” indicate significance and no significance, respectively, according to the LSD test at a 5% level of probability.

### 3.6 Effects of magnesium application on photosynthetic characteristics of rice flag leaf in rice at 20 days after heading

The impact of foliar application of different concentrations of magnesium sulfate on photosynthetic characteristics of flag leaf in rice 20 days after heading is shown in **Table 4**. As indicated in **Table 4**, the application of magnesium sulfate at different salinity levels significantly affected the photosynthetic characteristics of rice flag leaves, with a highly significant interaction effect on net photosynthetic rate, stomatal conductance, and transpiration rate. Significant differences in photosynthetic characteristics were observed among different concentrations of magnesium sulfate.

**Table 4:** Effects of magnesium application on photosynthetic characteristics of rice flag leaf at 20 days after heading.

Treatment		*P*n (μmol m^-2^ s^-1^)		*G*s (mol m^-2^ s^-1^)		*C*i (μmol m^-2^ s ^-1^)		*T*r (mmol m^-2^ s ^-1^)	
		2022	2023	2022	2023	2022	2023	2022	2023
S1	CK	12.05Eb	12.97Db	0.47Ea	0.36Da	361.18Aa	348.55Ab	3.92Ea	2.48Eab
	Mg1	19.19Bb	15.73Cc	0.80Ba	0.37Dc	332.82Ba	338.18Bb	4.90Ba	3.49Dc
	Mg2	20.92Ac	22.48Aa	0.84Aa	0.76Aa	323.27Da	324.86Ca	5.57Aa	5.24Aa
	Mg3	17.31Cb	18.29Ba	0.66Ca	0.61Ba	329.32Ca	335.53Ba	4.66Cb	4.71Ba
	Mg4	16.1Db	18.71Ba	0.56Da	0.45Cb	334.60Bb	336.38Bc	4.53Da	4.28Cb
S2	CK	15.34Ea	15.24Ba	0.34Eb	0.31Da	322.84Ac	349.80Ab	3.46Eb	2.93Da
	Mg1	19.77Ba	18.08Ab	0.53Bb	0.72Aa	310.47Db	337.79Bb	4.47Cc	5.01ABa
	Mg2	22.53Aa	19.02Ab	0.58Ab	0.74Ab	295.47Eb	313.42Ca	4.96Ac	5.13Aa
	Mg3	17.58Ca	19.36Aa	0.52Cb	0.65Ba	315.34Cb	337.17Ba	4.81Ba	4.87Ba
	Mg4	17.10Da	19.49Aa	0.44Db	0.56Ca	317.96Bc	342.05Bb	3.60Db	4.67Ca
S3	CK	10.15Ec	11.34Cc	0.20Ec	0.23Db	350.60Ab	354.86Aa	2.73Ec	2.04Cb
	Mg1	15.21Bc	20.31Aa	0.46Bc	0.63Bb	301.07Dc	347.88Ba	4.79Bb	4.10Ab
	Mg2	21.17Ab	19.29Ab	0.52Ac	0.68Ab	288.70Ec	324.15Da	5.13Ab	4.32Ab
	Mg3	14.76Cc	16.03Bb	0.41Cc	0.33Cb	330.64Ca	336.03Ca	4.66Cb	2.80Bb
	Mg4	11.76Dc	13.98BCb	0.25Dc	0.31Cc	338.96Ba	349.84Ba	3.29Dc	2.23Cc
F value	S	*		*		*		*	
	Mg	*		*		*		*	
	S×Mg	*		*		*		*	

*P*n, net photosynthetic rate; *G*s, stomatal conductance; *C*i, intercellular carbon dioxide concentration; *T*r, transpiration rate. S1, S2 and S3 represent mild, medium and severe saline-alkali soil, respectively. Mg1, Mg2, Mg3 and Mg4 represent 10, 20, 30 and 40 g L^-1^magnesium application, respectively. Within the same saline-alkaline soil, the values with different uppercase letters indicate significant differences in different magnesium application at a significance level of 5%; within the same magnesium application, the values with different lowercase letters indicate significant differences in different saline-alkaline soil at a significance level of 5%. “*” and “ns” indicate significance and no significance, respectively, according to the LSD test at a 5% level of probability.

In the two-year experimental data, compared with control, the application of magnesium sulfate significantly enhanced the net photosynthetic rate, stomatal conductance, and transpiration rate of rice leaves under different salinity levels, while significantly reducing the intercellular CO_2_ concentration. Comprehensive analysis of Mg treatment effects revealed that foliar magnesium sulfate supplementation significantly improved photosynthetic parameters in rice flag leaves compared to the control. In 2022, the net photosynthetic rate increased by an average of 52.5% (S1), 25.4% (S2), and 54.9% (S3), with further enhancements of 45% (S1), 24.6% (S2), and 53.5% (S3) in 2023. Stomatal conductance exhibited average increases of 53.2% (S1), 51.9% (S2), and 106.3% (S3) in 2022, followed by 53.7% (S1), 116% (S2), and 114.5% (S3) in 2023. Transpiration rate rose by 25.3% (S1), 29% (S2), and 63.5% (S3) in 2022, while in 2023, it showed more pronounced improvements of 78.5% (S1), 67.8% (S2), and 65.1% (S3). Conversely, intercellular CO_2_ concentration decreased by 8.6% (S1), 4% (S2), and 10.2% (S3) in 2022, with sustained reductions of 4.3% (S1), 4.9% (S2), and 4.3% (S3) in 2023.Significant differences in effects were observed among different concentrations of magnesium sulfate treatments, with the Mg2 treatment being the most effective. The overall performance over the two years was Mg2 > Mg1 > Mg3 > Mg4. The Mg2 treatment demonstrated the highest improvements in photosynthetic performance across both experimental years under all the four magnesium sulfate concentration treatments. Compared to those of control, the net photosynthetic rate of rice leaves increased by 73.6% and 73.3% (S1), 46.8% and 24.8% (S2), and 108.5% and 70.1% (S3) in 2022 and 2023, respectively. Stomatal conductance showed marked enhancements by 79.8% and 113.6% (S1), 70% and 141.6% (S2), and 164.1% and 201.4% (S3) in 2022 and 2023, respectively., Similarly, transpiration rate exhibited rises of 42.1% and 111.1% (S1), 43.4% and 75.1% (S2), and 87.8% and 112.3% (S3) in 2022 and 2023, respectively. In contrast, intercellular CO2 concentration displayed consistent reductions of 10.5% and 6.8% (S1), 8.5% and 10.4% (S2), and 17.7% and 8.7% (S3) in 2022 and 2023, respectively.

### 3.7 Effects of magnesium application on photosynthetic carbon assimilation and stomatal regulation in flag leaves of rice 20 days after heading

**Figure 4:**
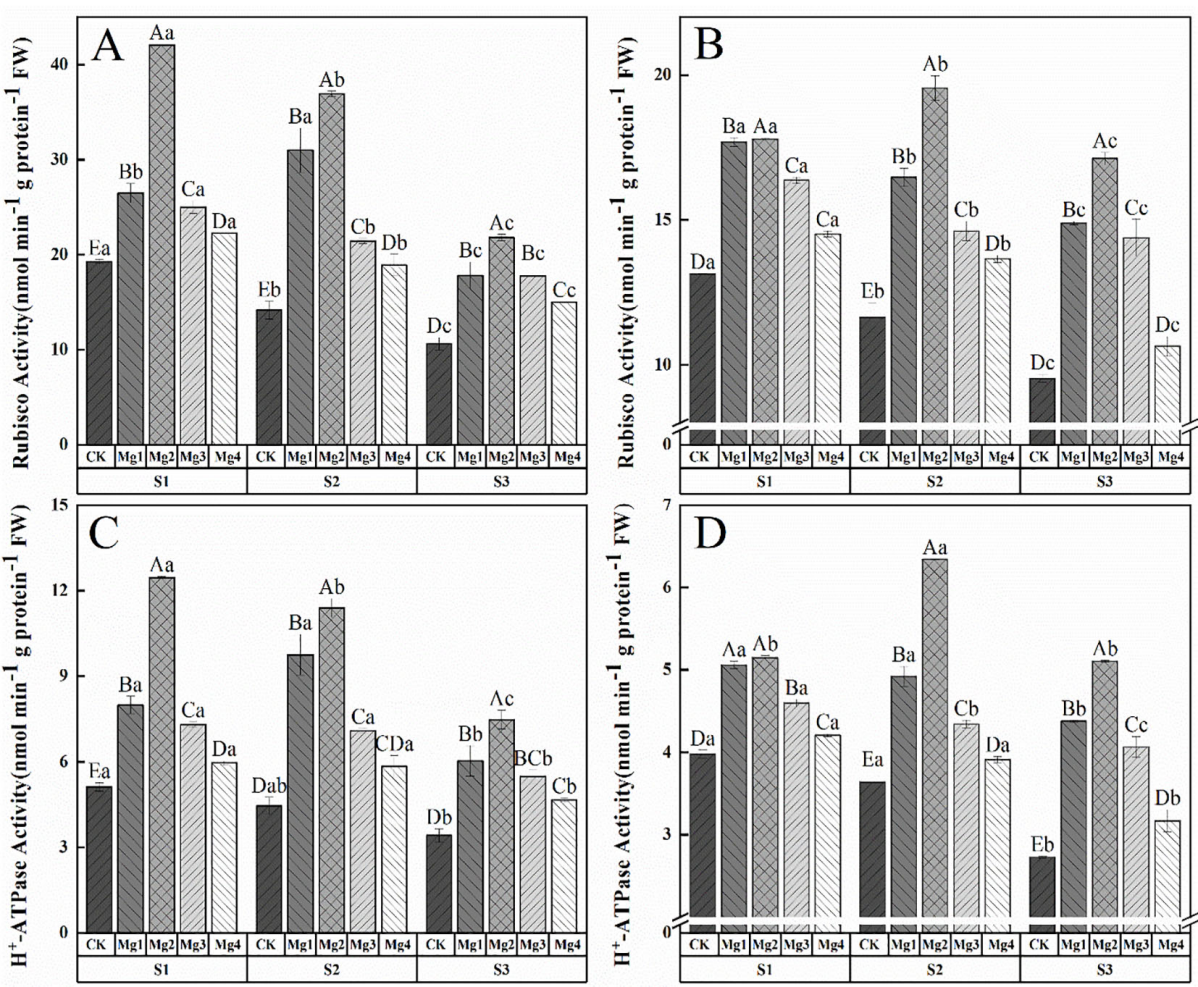
Effects of magnesium application on photosynthetic carbon assimilation and stomatal regulation in flag leaves of rice 20 days after heading. S1, S2 and S3 represent mild, medium and severe saline-alkali soil, respectively. Mg1, Mg2 Mg3 and Mg4 represent 10, 20, 30 and 40 g L^-1^magnesium application, respectively. Rubisco enzyme activity of 2022 and 2023 **(A, B)**, H^+^-ATP enzyme activity of 2022 and 2023 **(C, D)**. Within the same saline-alkaline soil, the values with different uppercase letters indicate significant differences in different magnesium application at a significance level of 5%; within the same magnesium application, the values with different lowercase letters indicate significant differences in different saline-alkaline soil at a significance level of 5%.

As shown in **Figure 4**, the application of magnesium sulfate at different concentrations significantly increased the activity of Rubisco in rice flag leaves 20 days after heading under different saline-alkaline soils in both years of the study. The effects varied significantly among different magnesium sulfate treatments, with the enhancement being Mg2 > Mg1 > Mg3 > Mg4 > CK. The Mg2 treatment yielded the best results, showing increases of 118.4% and 35.5% (S1), 160.8% and 68% (S2), 104.9% and 79.6% (S3) compared to the control in the two years, respectively. As shown in **Figure 4**, foliar magnesium sulfate application differentially modulated H^+^-ATPase activity and Rubisco carboxylation in rice flag leaves, with Mg2 treatment exhibiting the most pronounced effects. Under high salinity (S3), Mg2 elevated H^+^-ATPase activity by 119.1% (2022) and 87.3% (2023), which mechanistically linked to enhanced stomatal conductance (Gs improved on average by 177.8%) and Na^+^/K^+^ homeostasis. This proton-pumping activity energized guard cell turgor, facilitating CO_2_ influx and synergistically amplifying Rubisco activity and net photosynthetic rate. Similar trends were observed in S1 as elevated H^+^-ATPase activity by 143.6% (2022) and 29.4% (2023) also and S2 as 156% (2022) and 74.4% (2023). Though low-salinity conditions (S1) showed attenuated physiological gains due to redundant Mg²^+^ uptake and lower oxidative stress.

### 3.8 Effects of magnesium application on indicators of oxidative damage and osmotic homeostasis in rice leaves at 20 days after heading

As analyzed in **Table 5**, there was a highly significant interaction effect between different concentrations of magnesium sulfate and salt treatments on the content of osmoregulatory substances in rice flag leaves 20 days post-heading. In the 2022–2023 trials, the contents of soluble sugars and proline in the flag leaves 20 days post-heading showed a trend of first increasing and then decreasing with the increase of magnesium sulfate concentrations, with the maximum values generally observed under the S3 treatment. The contents of soluble proteins, malondialdehyde, and hydrogen peroxide, as peroxidation products, generally increased with the rise in salt concentrations. The treatment effects among different concentrations of magnesium sulfate were significantly different, with Mg2 showing the most optimal effect overall. Over the two years of trials, compared to the control treatment, the Mg2 treatment increased the content of soluble sugars by 96.8% and 46.6% (S1), 30% and 15.8% (S2), 55.6% and 86.2% (S3); proline content by 15.5% and 12.5% (S1), 16.5% and 25.3% (S2), 20.8% and 35% (S3); soluble protein content decreased by 50.9% and 13.7% (S1), 54.8% and 26.8% (S2), 32.9% and 31.4% (S3); malondialdehyde content decreased by 70.6% and 48.2% (S1), 48.3% and 43.6% (S2), 80% and 44.1% (S3); hydrogen peroxide content decreased by 49.5% and 32.3% (S1), 35.5% and 36.6% (S2), 39.5% and 59.6% (S3).

**Table 5:** Effects of magnesium application on indicators of oxidative damage and osmotic homeostasis in rice leaves at 20 days after heading.

Treatment		SP (mg g^-1^ FW)		SS (mg g^-1^ FW)		Pro (μg g^-1^ FW)		MDA (nmol g^-1^ FW)		H_2_O_2_ (μmol mg^-1^ FW)	
		2022	2023	2022	2023	2022	2023	2022	2023	2022	2023
S1	CK	35.66Ab	49.85Ab	3.91Eb	11.60Dc	40.28Dc	31.11Dc	52.47Ac	24.74Aa	20.59Aa	15.81Ac
	Mg1	27.02Dab	41.93Eb	7.15Bc	16.34Bb	44.99ABa	33.91Bc	31.54Ca	16.81Da	13.14Da	11.36Cb
	Mg2	17.52Ec	43.02Da	7.69Ab	17.00Ab	46.50Ab	34.99Ac	15.43Eb	12.82Ea	10.41Eb	10.71Cb
	Mg3	28.95Cb	44.50Cb	5.48Cc	16.07Bb	43.76BCb	32.51Cc	26.08Db	18.50Ca	16.52Ca	11.71BCc
	Mg4	31.41Bb	48.28Bb	4.47Dc	14.30Cb	42.70Cb	31.43Dc	39.39Ba	20.26Ba	17.64Ba	12.83Bb
S2	CK	43.64Aab	54.27Ab	6.29Ea	14.90Da	42.02Cb	33.66Cb	63.56Ab	23.18Aa	18.41Ab	21.60Ab
	Mg1	23.36Cb	43.21Cb	10.67Aa	16.59Bb	48.40Aa	40.46ABb	25.41Da	13.50Ca	11.42Dc	14.51Ca
	Mg2	19.74Cb	39.74Db	8.18Bb	17.24Ab	48.95Ab	42.19Ab	32.87Ca	13.08Ca	11.87Dab	13.70Ca
	Mg3	30.48Bb	47.86Bab	7.69Ca	16.40Bb	45.45Bb	38.43Bb	35.62Ca	13.89Cb	15.57Ca	15.02Ca
	Mg4	33.88Bb	50.20Bb	6.94Da	15.92Ca	44.31BCb	37.21Bb	46.33Ba	19.88Ba	16.53Ba	18.50Ba
S3	CK	54.25Aa	65.19Aa	5.88Ea	13.69Eb	44.59Ca	40.50Da	73.45Aa	24.08Aa	20.83Aa	26.69Aa
	Mg1	35.70BCa	48.88CDa	8.30Bb	18.66Ca	48.80Ba	50.34Ba	28.01Ca	14.80Ba	12.13Cb	12.08BCb
	Mg2	36.40Ca	44.74Da	9.14Aa	25.49Aa	53.85Aa	54.66Aa	14.65Eb	13.46Ca	12.59Ca	10.78Cb
	Mg3	30.75BCa	50.33Ca	6.69Cb	21.28Ba	51.59Aa	47.86Ba	21.77Db	13.60Cb	12.99Cb	12.72Bb
	Mg4	40.47Ba	60.45Ba	6.22Db	15.76Da	48.88Ba	44.46Ca	43.44Ba	15.04Bb	16.81Ba	12.3BCb
F value	S	*		*		*		*		*	
	Mg	*		*		*		*		*	
	S×Mg	*		*		*		*		*	

SP, soluble protein; SS, soluble sugar; Pro, proline; MDA, malondialdehyde; H_2_O_2_, hydrogen peroxide. S1, S2 and S3 represent mild, medium and severe saline-alkali soil, respectively. Mg1, Mg2 Mg3 and Mg4 represent 10, 20, 30 and 40 g L^-1^magnesium application, respectively. Within the same saline-alkaline soil, the values with different uppercase letters indicate significant differences in different magnesium application at a significance level of 5%; within the same magnesium application, the values with different lowercase letters indicate significant differences in different saline-alkaline soil at a significance level of 5%. “*” and “ns” indicate significance and no significance, respectively, according to the LSD test at a 5% level of probability.

### 3.9 Effect of magnesium application on the antioxidant system of flag leaves in rice 20 days after heading

**Figure 5:**
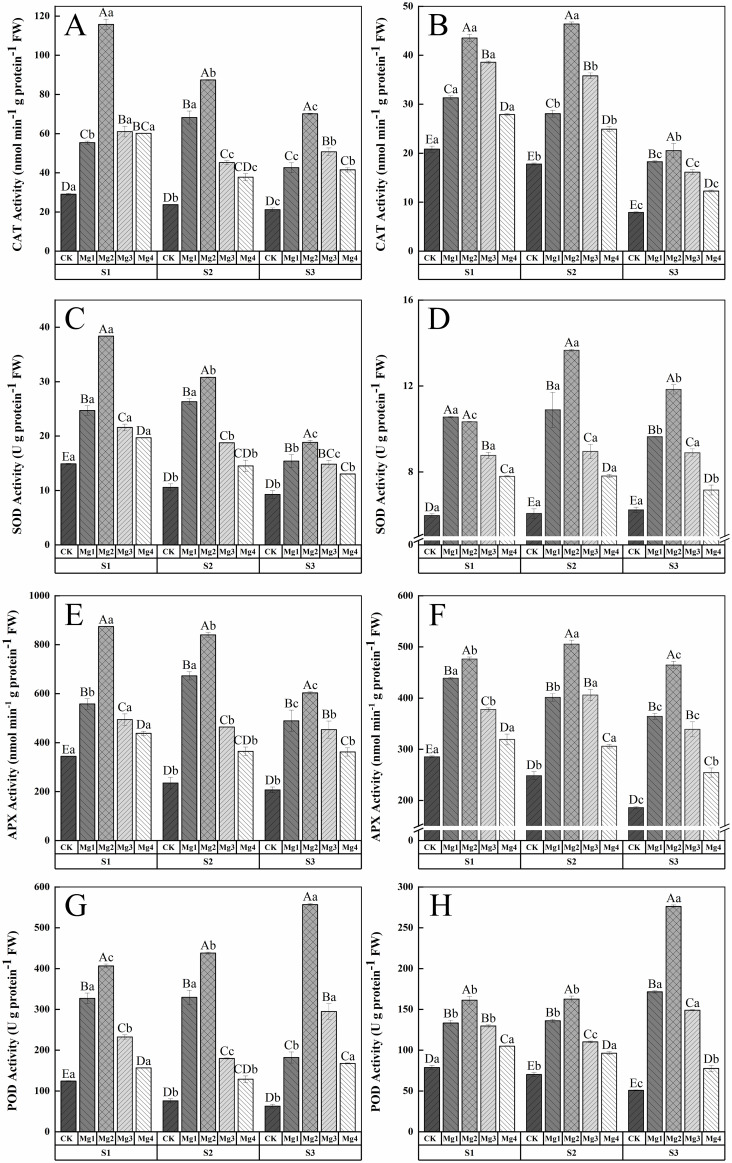
Effect of magnesium application on the antioxidant system of flag leaves in rice 20 days after heading. S1, S2 and S3 represent mild, medium and severe saline-alkali soil, respectively. Mg1, Mg2 Mg3 and Mg4 represent 10, 20, 30 and 40 g L^-1^magnesium application, respectively. CAT enzyme activity of 2022 and 2023 **(A, B)**, SOD enzyme activity of 2022 and 2023 **(C, D)**, APX enzyme activity of 2022 and 2023 **(E, F)**, POD enzyme activity of 2022 and 2023 **(G, H)**, Within the same saline-alkaline soil, the values with different uppercase letters indicate significant differences in different magnesium application at a significance level of 5%. Within the same magnesium application, the values with different lowercase letters indicate significant differences in different saline-alkaline soil at a significance level of 5%.

As shown in **Figure 5**, the application of magnesium sulfate at different concentrations significantly increased the CAT enzyme activity in rice leaves 20 days after heading in both years of the study. The enhancement of rice leaf resistance varied with the concentration of magnesium sulfate, showing significant differences among treatments. Overall, the Mg2 treatment was the most effective across different salt concentrations, significantly increasing by 299.5%and 109.1%(S1), 269.6%, and 161%(S2), 232.2%and 160.4%(S3) compared to the control (CK) in 2022-2023, respectively. **Figure 5** indicate that the SOD enzyme activity in rice leaves initially increased and then decreased with the rise in magnesium sulfate concentration over the two years, with the treatment effect ranking as Mg2 > Mg1 > Mg3 > Mg4 > CK. In 2022, the maximum SOD activity of 38.35 U g protein^-1^ FW was achieved under S1 treatment, a157.9% increase compared to the CK. In 2023, the maximum activity of 13.66 U g protein^-1^ FW was reached under S2 treatment, a 125.6% increase over the CK. **Figure 5** show that the APX enzyme activity in rice leaves under different salt concentrations was significantly enhanced by magnesium sulfate treatments, with significant differences among treatments. In 2022, the Mg1 treatment increased APX activity by 62.3% (S1), 186.9% (S2), and 136.6% (S3) compared to the control, while the Mg2 treatment increased it by 154.4% (S1), 258.4% (S2), and 191.9% (S3). The Mg3 and Mg4 treatments showed increases of 43.7% (S1), 97.6% (S2), 119.2% (S3) and 27.3% (S1), 55.4% (S2), 75% (S3), respectively. In 2023, the Mg1, Mg2, Mg3, and Mg4 treatments increased APX activity by 53.8% (S1), 61.7% (S2), 95.9% (S3), 67% (S1), 103.6% (S2), 149.9% (S3), 32.4% (S1), 63.5% (S2), 82.3% (S3), and 11.9% (S1), 23.1% (S2), 36.8% (S3), respectively. **Figure 5** illustrate that the POD enzyme activity in untreated rice leaves decreased with increasing salt concentration, while magnesium sulfate treatments significantly enhanced POD activity. The overall trend was similar to that of SOD activity, initially increasing and then decreasing with the rise in magnesium sulfate concentration, with the Mg2 treatment being the most effective. The maximum POD activity of 556.7 U g protein^-1^ FW and 276.1 U g protein^-1^ FW was achieved under S3 treatment in both years, representing increases of 791.2% and 446.2% over the control, respectively.

### 3.10 Effects of magnesium application on element contents in various organs of rice at maturity stage

**Figure 6:**
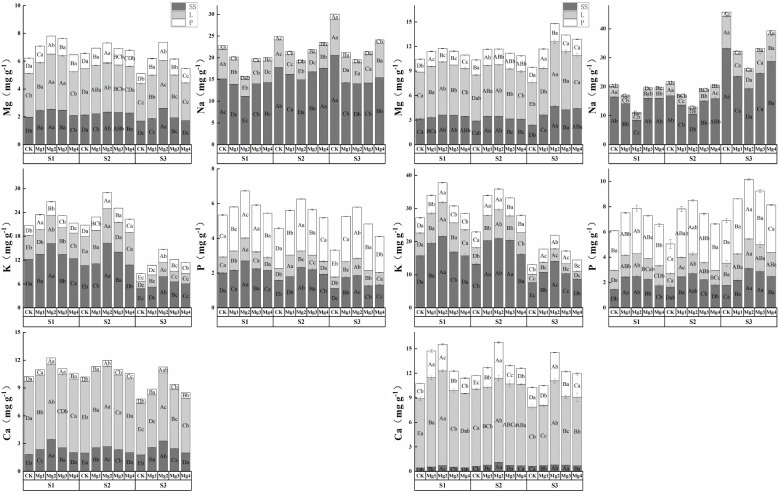
(1) Effects of magnesium application on elemental content in various organs of rice at maturity stage in 2022. SS represents stem sheath; L represents leave; P represents panicle. S1, S2 and S3 represent mild, medium and severe saline-alkali soil, respectively. Mg1, Mg2 Mg3 and Mg4 represent 10, 20, 30 and 40 g L-1magnesium application, respectively. Within the same saline-alkaline soil, the values with different uppercase letters indicate significant differences in different magnesium application at a significance level of 5%; within the same magnesium application, the values with different lowercase letters indicate significant differences in different saline-alkaline soil at a significance level of 5%. (2) Effects of Magnesium Application Elemental Content in Various Organs of Rice at Maturity Stage in 2023. In the figure, SS represents stem sheath; L represents leave; P represents panicle. S1, S2 and S3 represent mild, medium and severe saline-alkali soil, respectively. Mg1, Mg2 Mg3 and Mg4 represent 10, 20, 30 and 40 g L-1magnesium application, respectively. Within the same saline-alkaline soil, the values with different uppercase letters indicate significant differences in different magnesium application at a significance level of 5%; within the same magnesium application, the values with different lowercase letters indicate significant differences in different saline-alkaline soil at a significance level of 5%.

In the experiments conducted in 2022 (**Figures 6-1**) and 2023 (**Figures 6-2**), the elemental content in various organs of rice at maturity varied significantly. The distribution of Mg content was highest in leaves, followed by stems and sheaths, and then panicle; the distribution of Na and K content was highest in stems and sheaths, followed by leaves, and then panicle; the distribution of P content was highest in panicle, followed by stems and sheaths, and then leaves; and Ca was predominantly found in leaves. As the salinity concentration increased, the contents of Mg, K, P, and Ca in the stems, sheaths, and leaves of rice at maturity showed a gradual decline, while the contents of Mg, K, P, and Ca in the panicle first increased and then decreased, and the content of Na increased with the rise in salinity concentration. In both experimental years, the Mg content in different parts of the rice at maturity reached its maximum under the Mg2 treatment, with stems and sheaths content increasing by an average of 44.3% and 31.3% compared to the control treatment, leaf content increasing by an average of 25.6% and 19.8%, and panicle content increasing by an average of 45.6% and 9.3%. Similarly, Na content reached its minimum under this treatment, with stems and sheaths content decreasing by an average of 24.8% and 42.9% compared to the control treatment, leaf content decreasing by an average of 40.2% and 43.2%, and panicle content decreasing by an average of 35.8% and 36.1%. The application of magnesium sulfate at varying concentrations significantly enhanced the elemental content in various organs of rice at maturity, with significant differences among treatments. Overall, except for the Na element, which initially decreased and then increased, the content of other elements generally increased first and then decreased with the increase in magnesium sulfate concentration. The effectiveness of different concentrations of magnesium sulfate treatments was ranked as Mg2 > Mg1 > Mg3 > Mg4.

### 3.11 Effects of magnesium application on element ratios in various organs of rice at maturity stage

**Figure 7:**
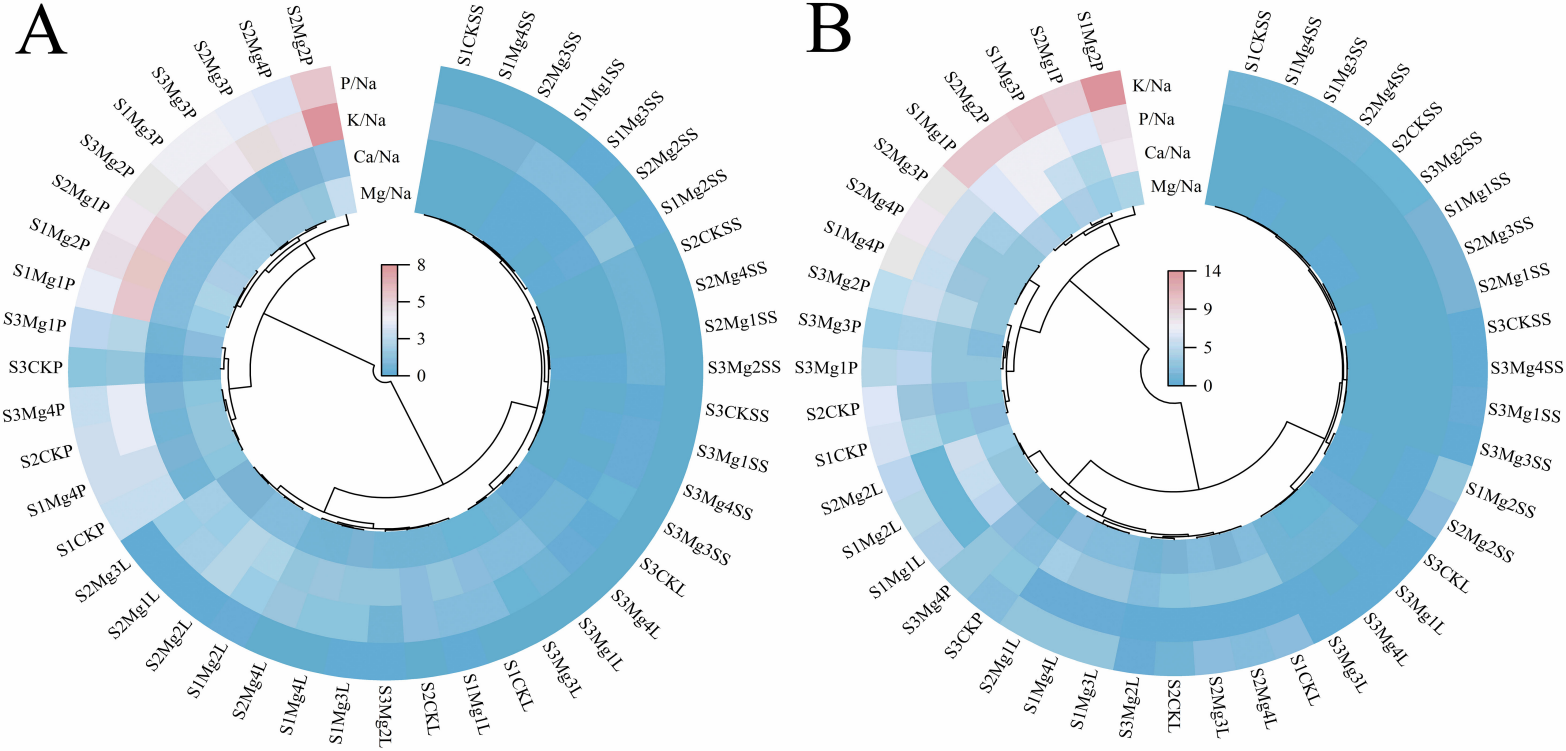
Analysis of elemental ratio heat maps in various organs of rice at maturity stage of magnesium application. **(A)** shows the year 2022 and **(B)** shows the year 2023. S1, S2 and S3 represent mild, medium and severe saline-alkali soil, respectively. Mg1, Mg2 Mg3 and Mg4 represent 10, 20, 30 and 40 g L^-1^magnesium application, respectively. MS is expressed as the Maturity stage; SS represents stem sheath; L represents leave; P represents panicle.

Analysis of **Figure 7** indicates that, overall, during the two-year experiment, the ratios of Mg/Na, K/Na, P/Na, and Ca/Na in various organs of rice at maturity decreased with increasing salt concentration. Notably, the decline in ion ratios was more pronounced in the leaves compared to other parts. Following the application of different concentrations of exogenous magnesium sulfate, the ion ratios in the rice organs initially increased and then decreased as the concentration of exogenous magnesium sulfate rose, with the highest values observed in the spike under the Mg2 treatment. The effectiveness of exogenous magnesium sulfate treatments was ranked as follows: Mg2 > Mg1 > Mg3 > Mg4 > CK, with the ion ratios in rice organs exhibiting the order: panicle > leaves > sheath.

### 3.12 Effects of magnesium application on economic benefits of rice production

As shown in **Table 6**, across the two-year field trials, rice earnings peaked at S1 and gradually decreased as salt concentration increased. Foliar application of magnesium sulfate significantly increased rice earnings, with the Mg2 treatment consistently outperforming other treatments under different saline-alkali soil conditions. Compared to the control treatment, the Mg2 treatment achieved a maximum rice revenue of 11,825 CNY ha^-^¹, representing a 33.9% increase.

**Table 6:** Effects of magnesium application on economic benefits of rice production.

Treatment		Value of rice production (× 10^3^ CNY ha^-1^)		Mechanical operating costs (CNY ha^-1^)		Labor cost (CNY ha^-1^)		Fertilizer (CNY ha^-1^)		Magnesium sulfate fertilizer (CNY ha^-1^)		Total cost (CNY ha^-1^)		Earnings (CNY ha^-1^)	
		2022	2023	2022	2023	2022	2023	2022	2023	2022	2023	2022	2023	2022	2023
S1	CK	22.8Ca	19.8Ca	5148.2		1400		7420.8		0		13968.9		8831.1Ca	5831.1Da
	Mg1	24Ba	22.5Ba	5148.2		1400		7420.8		3		13971.9		10028ABa	8528.1Ba
	Mg2	25.8Aa	23.4Aa	5148.2		1400		7420.8		6.1		13975		11825.0Aa	9425.0Aa
	Mg3	23.4Ba	20.4Ca	5148.2		1400		7420.8		9.1		13978		9422.0Ba	6422.0Ca
	Mg4	22.2Ca	20.1Ca	5148.2		1400		7420.8		12.2		13981.1		8218.9Da	6118.9CDa
S2	CK	17.7Db	15.3Db	5148.2		1400		7420.8		0		13968.9		3731.1Eb	1331.1Eb
	Mg1	21.3Bb	19.5Cb	5148.2		1400		7420.8		3		13971.9		7328.1Cb	5528.1Cb
	Mg2	23.1Ab	22.2Ab	5148.2		1400		7420.8		6.1		13975		9125.0Ab	8225.0Ab
	Mg3	21.9Bb	21Bb	5148.2		1400		7420.8		9.1		13978		7922.0Bb	7022.0Bb
	Mg4	19.2Cb	18.6Cb	5148.2		1400		7420.8		12.2		13981.1		5218.9Db	4618.9Db
S3	CK	15Dc	13.5Dc	5148.2		1400		7420.8		0		13968.9		1031.1Dc	-468.9Dc
	Mg1	19.2Bc	18.6Bc	5148.2		1400		7420.8		3		13971.9		5228.1Bc	4628.1Bc
	Mg2	20.4Ac	21Ac	5148.2		1400		7420.8		6.1		13975		6425.0Ac	7025.0Ac
	Mg3	18.3Cc	18.6Bc	5148.2		1400		7420.8		9.1		13978		4322.0Cc	4622.0Bc
	Mg4	15.3Dc	17.1Cc	5148.2		1400		7420.8		12.2		13981.1		1318.9Dc	3118.9Cc
F value	S	*		–		–		–		–		–		*	
	Mg	*		–		–		–		–		–		*	
	S×Mg	*		–		–		–		–		–		*	

S1, S2 and S3 represent mild, medium and severe saline-alkali soil, respectively. Mg1, Mg2 Mg3 and Mg4 represent 10, 20, 30 and 40 g L^-1^magnesium application, respectively. CNY represent Chinese Yuan. Rice paddy price represent 3 CNY kg^-1^, Magnesium sulfate fertilizer represent 800 CNY t^-1^. Within the same saline-alkaline soil, the values with different uppercase letters indicate significant differences in different magnesium application at a significance level of 5%; Within the same magnesium application, the values with different lowercase letters indicate significant differences in different saline-alkaline soil at a significance level of 5%. “*” and “ns” indicate significance and no significance, respectively, according to the LSD test at a 5% level of probability.

## 4 Correlation analysis

**Figure 8:**
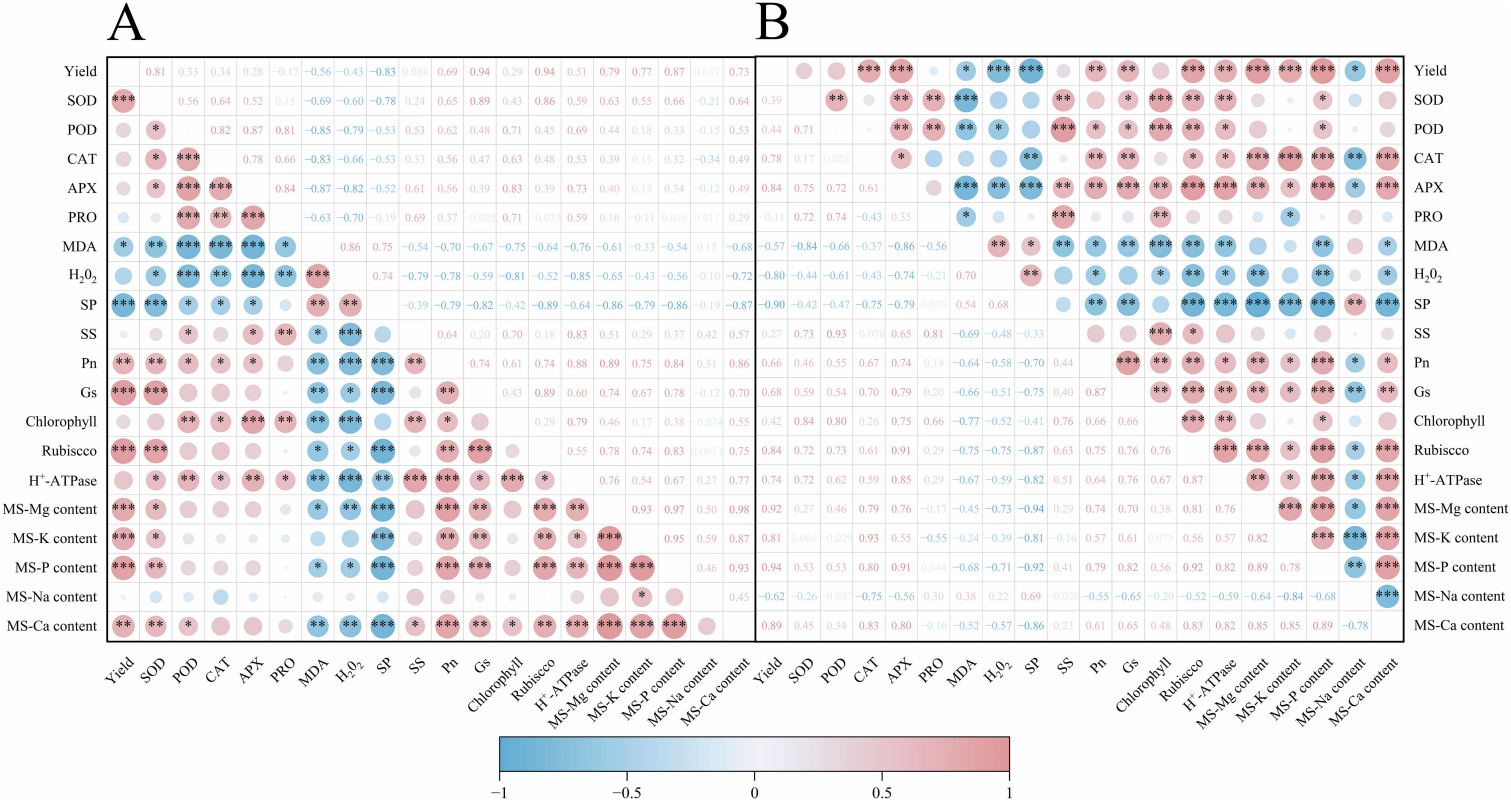
Effects of magnesium application on rice yield and correlation analysis of related indicators in 2022-2023. **(A)** shows the year 2022 and **(B)** shows the year 2023. SP refers to soluble protein; SS denotes soluble sugar; *P*n represents the net photosynthetic rate; *G*s denotes the stomatal conductance; MS-Mg content represents the magnesium content at maturity stage; MS-K content denotes the potassium content at maturity stage; MS-P content signifies the phosphorus content at maturity stage; MS-Na content indicates the sodium content at maturity stage; MS-Ca content refers to the calcium content at maturity stage. "*" denotes a significant interaction at the 0.05 level; "**" denotes a significant interaction at the 0.01 level; "***" denotes a significant interaction at the 0.001 level.

As shown in **Figures 8**, upon conducting a correlation analysis of rice yield and related physiological indicators across two years of trials, it was observed that rice yield exhibited a significant positive correlation with the activities of antioxidant enzymes such as CAT and POD, the activity of photosynthetic assimilatory enzymes like Rubisco, and the content of mature-stage nutrients including Mg, K, P, and Ca, with correlation coefficients greater than 0.5 (**Figure 8**). Conversely, there was a significant negative correlation with correlation coefficients less than -0.5 between rice yield and leaf membrane lipid peroxidation products MDA and H_2_O_2_, soluble protein, and the content of toxic sodium ions at maturity. Additionally, it was noted that net photosynthetic rate and chlorophyll content had varying impacts on the physiological indicators of rice leaves.

## 5 Discussion

Over the past decade, rice has been recognized as a pioneer crop for saline-alkali soil reclamation, and the impacts of salt stress on its yield formation process, along with the underlying mechanisms, have been extensively studied and well-documented (Leung et al., 2002; Ganie et al., 2019; Jiang et al., 2024). Studies by Wei (Wei et al., 2024) and Geng (Geng et al., 2024) have demonstrated that under saline-alkaline conditions, key indicators such as the number of grains per panicle, 1000-grain weight, and seed setting rate in rice significantly decrease, collectively leading to a substantial reduction in rice yield. In the current study, rice yield in severe saline-alkaline soil was approximately 33.3% and 31.8% lower than that in median saline-alkaline soil over two years. Compared to that in 2022, rice yield under varying salt concentrations in 2023 declined by 12% (S1), 13.6% (S2), and 10% (S3), respectively. It showed that all components of rice yield are affected by salt stress, leading to a significant decline in yield. In the current study, magnesium sulfate sparaying displayed significant improvements in rice grain filling rate and thousand-grain weight and increased rice yield by an average of 6.7% (S1), 27.3% (S2), and 30.2% (S3) across the two years, whereas Mg2 treatment showed the most significant yield increasing effect among all the four application rates. The difference in the yield improvement effect of magnesium sulfate application on rice under different salt concentrations originates from the threshold-dependent interaction between magnesium-mediated stress tolerance and the plant’s physiological requirements. Under the high-salinity condition (S3: 4.7 mS cm^-1^), Mg^2+^ treatment significantly alleviated Na^+^ toxicity by reducing Na^+^ accumulation in the aboveground parts, promoting Mg^2+^ absorption, and enhancing chloroplast integrity. Meanwhile, Mg^2+^ synergistically activated the antioxidant network, systematically reducing oxidative damage and improving photosynthetic carbon assimilation efficiency. These reparative effects facilitated the allocation of dry matter to the grains, increasing the filled-grain percentage and achieving an average yield increase of 30.2%. Under the low-salinity condition (S1: 2.54 mS cm^-1^), the effect of Mg^2+^ sparying was weakened in some degree due to the relatively lower Na^+^ stress accompanied with higher aboveground Mg^2+^ level compared to those under higher salinity stress. Despite minor improvements in the antioxidant system and leaf pigments, photosynthesis approaching saturation and nutrient redundancy resulted in a small yield increase in rice. This result was consistent with the findings of Hao’s research (Hao, 2023), whereas both studies did not provide a detailed analysis of the specific mechanisms by which Mg^2+^ alleviate Na^+^ accumulation, improve antioxidant systems, and enhance photosynthesis.

Magnesium, as an essential macronutrient in photosynthesis, plays a crucial role in maintaining carbohydrate synthesis and promoting the transport between “source and sink,” thereby enhancing crop yield and quality, such as in cowpea (Ciscomani-Larios et al., 2021), sugar beet (Pogłodziński et al., 2021), tobacco (Ishfaq et al., 2022), and tea (Ruan et al., 2011). Previous studies have shown that exogenous magnesium fertilizer effectively enhances biomass accumulation and chlorophyll content in timothy grass (Radkowski et al., 2017) and faba beans (Neuhaus et al., 2014).It has also been demonstrated to improve carbohydrate and nutrient distribution (nitrogen, phosphorus, potassium) in bananas under nutrient-deficient conditions (He et al., 2020).In wheat, magnesium application significantly increases photosynthetic system efficiency and promotes carbohydrate transport (Ba et al., 2020).Additionally, magnesium elevates antioxidant enzyme activity in pepper (Sevgin Zirek and Uzal, 2020) and rice seedlings (Xuan et al., 2022) exposed to salt stress. These combined effects ultimately promote normal plant growth under various abiotic stresses. In this study, we revealed that the physiological mechanisms by which foliar application of magnesium sulfate alleviates salt stress in rice are salt-dependent. Under conditions of severe salinity and alkalinity(S3), the application of an appropriate amount of magnesium sulfate via leaf spraying resulted in an average increase of 45.4% in chlorophyll a content, optimizing the chlorophyll a/b ratio. This is attributed to magnesium ions serving as the central metal ion in the chlorophyll porphyrin ring, where their concentration directly influences the pigment content and photosynthetic capacity of the leaves. The effective supplementation of exogenous magnesium ions may have inhibited the expression of the *SGR* gene, reduced the activity of de-magnesium chlorophyllase, delayed chlorophyll degradation, and maintained the thylakoid pH gradient and membrane potential. This ensured the smooth transfer of electrons from PSII to PSI, preventing the formation of triplet state chlorophyll, thereby reducing the generation of singlet oxygen (¹O_2_) at its source (Meng et al., 2023). Research indicates that the *OsMGT3* magnesium transporter on the chloroplast membrane facilitates the import of exogenous Mg²^+^ from the cytoplasm into the chloroplast, allowing the lysine residues at the active site of the Rubisco large subunit to effectively bind with Mg²^+^ and CO_2_, forming the Rubisco-Mg²^+^-CO_2_ complex, which maintains the magnesium ion concentration required for Rubisco activation (Chen et al., 2022). Furthermore, the exogenous magnesium sulfate indirectly prevents the oxidative modification of the Rubisco large subunit by reducing ROS production at its source, thereby preserving its structural stability and enzymatic activity. This aligns with our experimental observations, which showed an approximate 92.2% increase in Rubisco enzyme activity and an approximately 89.3% increase in net photosynthetic rate compared to the control treatment. However, since the gene expression or protein abundance related to pigment synthesis, degradation, and Rubisco activation were not determined in the current study, further molecular biology experiments are necessary to validate the inferred mechanisms regarding the delay in chlorophyll synthesis and the maintenance of Rubisco function. In studies involving leguminous plants, the application of exogenous magnesium sulfate has been found to significantly enhance the expression of the H^+^-ATPase gene (primarily *vha2*). This activation aids in enhancing proton pump activity, promoting the transmembrane transport of other metabolic products such as sucrose, and providing dynamic support for the distribution of photosynthetic products (Neuhaus et al., 2013). In our experiment, H^+^-ATPase activity increased by approximately 119.1%, which may correlate with these findings. Numerous studies have demonstrated that magnesium ions can enhance cell membrane integrity, mitigate ROS accumulation induced by salt stress, reduce the stress load on the antioxidant system, and improve enzyme activity stability. As a cofactor or structural stabilizer for enzymes, magnesium may directly participate in maintaining the conformation and catalytic activity of antioxidant enzymes under optimal pH and redox conditions. Although there is currently a lack of conclusive molecular evidence regarding the direct regulation of antioxidant enzyme gene expression by Mg²^+^, it is possible that it indirectly promotes their transcription or translation by influencing ROS-mediated signaling pathways (such as *Ca²^+^/MAPK*). This is also reflected in our experiment, where antioxidant enzyme activity was significantly enhanced compared to the control treatment. However, the lack of analysis regarding the changes in the expression of ROS signaling pathways and antioxidant enzyme-encoding genes means that the signal regulation inferred from the literature still requires molecular evidence support.

In contrast, under mild saline-alkali soil (S1), although magnesium sulfate application also increased chlorophyll a content (by approximately 58.2%) and antioxidant enzyme activity, the mild oxidative stress (with H_2_O_2_ content decreasing by approximately 32.3%) and relatively stable ion balance resulted in diminished physiological benefits from exogenous magnesium sulfate. Compared to high salinity conditions, the redundant absorption of nutritional elements in rice leaves and stems at low salinity concentrations failed to surpass the photosynthetic saturation threshold, indicating a diminishing marginal benefit of the antioxidant system and ion homeostasis investment. Overall, the treatment effects at concentrations of 10, 20, 30, and 40 g L^-1^ showed a decreasing trend with increasing magnesium sulfate concentration. This indicates that excessive magnesium sulfate fertilizer negatively impacts rice growth, yield, and physiological responses, consistent with findings by Suman (Lamichhane et al., 2023) although it still improved rice growth to some extent in saline-alkali soil.

Research has indicated that under salt stress, rice can synthesize soluble organic osmotic adjustment substances to alter cell osmotic potential, thereby alleviating physiological drought caused by the accumulation of high concentrations of sodium ions (Rao et al., 2013). Proline, as an osmotic pressure-regulating solute, can stabilize cell structures and maintain organelle functionality under stress conditions such as salt stress to support normal plant growth; sugars, as the final products of photosynthetic assimilation, together with proline, participate in the defense mechanism against abiotic stress in rice (Alavilli et al., 2023; Zhang et al., 2023). In this experiment, the contents of proline and soluble sugar in rice leaves both gradually increased with the increase in salt concentration. Moreover, the levels of both substances showed a trend of first increasing and then decreasing as the concentration of magnesium sulfate increased, indicating that foliar application of magnesium sulfate, at appropriate concentrations, enhances the content of osmoregulatory substances in rice leaves, thereby alleviating the osmotic stress caused by sodium ions. With increasing salt concentrations, the accumulation of toxic sodium ions in various rice organs also increased, leading to a gradual disruption of ionic homeostasis, exacerbated physiological drought, and increased leaf tip necrosis; leaf cell membranes suffered more severe damage, with increased levels of peroxidative substances such as malondialdehyde and hydrogen peroxide. Foliar magnesium sulfate supplementation significantly enhanced chlorophyll content (a, b, and total) in rice flag leaves 20 days after heading, particularly under high salinity conditions (S3), where chlorophyll an increased by 59.8% compared to the control enhancing the net photosynthetic rate, increasing the synthesis and transport of photosynthates; it also elevated the activity of antioxidant enzymes and accumulated substantial osmoregulatory substances to maintain normal physiological and biochemical activities in the leaves. Foliar magnesium sulfate application alleviated salt stress-induced damage in rice, maintained high crop yields, and minimized losses in rice production on saline-alkali lands.

During the growth of rice, the absorption of various mineral elements is essential to maintain normal physiological and biochemical activities, as these elements play crucial roles in enzyme activity and metabolism (Zhao et al., 2004). However, numerous studies have shown that under saline-alkali stress, crops experience excessive accumulation of Na^+^, leading to a decline in the levels of essential nutrients such as K^+^, Ca^2+^, and Mg^2+^ within the plant. Additionally, the high concentration of Na^+^ competes for translocation sites on the cell membrane, significantly reducing the uptake of ions such as Mg^2+^ (Wang et al., 2011; Zhang, 2024). At the same time, the content and balance of mineral elements in plants vary significantly among different species and organs (Liu et al., 2017). In rice, Na^+^ primarily accumulates in the roots, with higher accumulation in the stem and sheath than in the leaves, which is a significant physiological mechanism for adapting to growth under saline-alkaline stress (Zhao et al., 2004). When the Na^+^ accumulation in leaves exceeds the cell compartmentalization capacity, it disrupts the plant’s internal ion homeostasis, leading to salt damage (Zhao et al., 2020). In this study, the Na content in various organs of rice at maturity was ranked as follows: stem and sheath > leaf > panicle. (**Figures 6-1, 2**), which is consistent with the findings of Jing and Zhang (Jing et al., 2017; Zhang, 2024). Under salt stress, Na^+^ primarily enters root cells passively through non-selective cation channels (*NSCCs*). Studies have shown that the application of exogenous Mg²^+^ can maintain membrane potential stability, reduce the passive leakage of Na^+^ through *NSCCs*, and may indirectly affect the activity of *OsHKT1;5* to decrease Na^+^ transport activity, thereby reducing Na^+^ accumulation and alleviating Na^+^ toxicity under salt stress, which enhances plant salt tolerance (Sevgin Zirek and Uzal, 2020). In experiments, the application of exogenous magnesium sulfate resulted in an average reduction of Na^+^ content in rice sheaths by 33.9%, in leaves by 41.7%, and in spikelets by 36% compared to the control. Concurrently, the plant’s ability to absorb Mg²^+^ was restored, likely relying on the activation of transport proteins *OsMGT1/OsMGT6*. High concentrations of Na^+^ competitively inhibit the activity of Mg²^+^ transport proteins, reducing magnesium absorption. After the application of exogenous magnesium sulfate, this process was partially reversed according to the law of mass action (Roy and Chowdhury, 2021). In the experiments, the Mg²^+^ content in rice leaves increased by approximately 22.7% (S1). Some studies suggest that Mg²^+^ may activate the *CBL-CIPK23* complex, which plays a crucial role in the regulation of Na^+^ and K^+^ transport, potentially also indirectly affecting the activity of *OsNHX1*, promoting Na^+^ compartmentalization and reducing Na^+^ toxicity in the cytoplasm (Pan et al., 2018). The *AtMHX* protein in vacuoles stores excess Mg²^+^, preventing Mg²^+^/Na^+^ imbalance in the cytoplasm, which aligns with the observation that the Mg²^+^/Na^+^ ratio under Mg²^+^ treatment was greater than that of the control (**Figure 7**). *AtMHX* also aids in storing cytoplasmic Mg²^+^ in vacuoles, maintaining the transmembrane driving force for potassium absorption (Chen et al., 2021), resulting in a 39.2% increase in potassium content in rice under Mg²^+^ treatment compared to the control (S1). Following the application of exogenous magnesium sulfate, the increased magnesium content in rice activated the phosphorylation of the Ca²^+^/H^+^ antiporter *OsCAX1*, which may synergistically promote the compartmental accumulation of Ca²^+^, aiding in cytosolic calcium homeostasis. Under high salt stress, high concentrations of Na^+^ occupy the Ca²^+^ binding sites of *CNGC*-type channels, leading to a decrease in the rate of calcium absorption. Experimental results indicated that the Ca²^+^ content in the S3 treatment decreased by approximately 14.8% compared to S1. Additionally, Na^+^ occupies the carboxyl groups of pectin, reducing the binding capacity of Ca²^+^, weakening the mechanical strength of the cell wall, and exacerbating ion leakage (Li et al., 2022). The application of exogenous magnesium sulfate may competitively bind to the EF-hand domain of *SOS3*, affecting its interaction with *SOS2*, thereby regulating the excessive activation of *SOS1* and preventing the collapse of the proton gradient. Furthermore, by synergistically enhancing the activity of antioxidant enzymes, it mitigates oxidative damage to Ca²^+^ channels (Yi et al., 2022), which is consistent with the observation that Ca²^+^ content under Mg²^+^ treatment increased by approximately 45.3% compared to the control (S1). However, due to the absence of key ion transport protein genes’ expression analysis (such as *OsHKT1;5*, *OsNHX1*, *OsMGT1*, and *OsCAX1*), the regulatory mechanisms of magnesium sulfate on rice ion balance remains at a hypothetical level.

It is noteworthy that in this experiment, the experimental site was located in a coastal saline-alkali land within 5 km of the sea, where the sandy loam soil texture and inherently high background levels of sodium (Na^+^) and potassium (K^+^) collectively resulted in significantly reduced magnesium (Mg²^+^) availability. This substantially diminished rice uptake and utilization of soil Mg²^+^, despite its total content falling within the normal range (Yan and Hou, 2018). Ion analysis further revealed that the application of exogenous magnesium sulfate (MgSO_4_) did not reduce rice uptake of other essential nutrients (e.g., P, K^+^, Ca^2+^), thereby excluding potential ionic interference effects. Despite a series of significant physiological responses were obtained in this study, the inability to establish a complete control treatment (no salt + no magnesium sulfate application) due to field trial conditions somewhat limits our analysis of the independent effects of each treatment. Besides, assessments of related gene expression or protein abundance were lacked regardless of enzyme activity, ion content, and photosynthetic parameters. This has led to a mechanism analysis that is largely based on literature speculation, lacking direct molecular evidence, which presents several limitations in our experimental research. And the same time the application of magnesium sulfate via foliar spraying not only supplemented the exogenous magnesium sulfate for rice grown in saline-alkali soils but also introduced SO_4_²^-^, which is an important component of sulfur assimilation and tolerance to abiotic stress. Previous studies have indicated that appropriate concentrations of SO_4_²^-^ can enhance the antioxidant capacity of crops through the synthesis of glutathione (Barlas et al., 2023). The concentrations of magnesium sulfate applied in this study were 10 g L^-1^, 20 g L^-1^, 30 g L^-1^, and 40 g L^-1^, corresponding to sulfur contents ranging from 1.3 g L^-1^ to 5.2g L^-1^. This is significantly higher than the recommended suitable concentrations reported in earlier studies (4.66–9.32 mg L^-1^) (Sumardiyono et al., 2015). This suggests that SO_4_²^-^ may partially contribute to alleviating oxidative stress, while our data strongly support that Mg²^+^ is the primary driving force behind the observed phenomena.

## 6 Conclusions

This study pioneers the investigation of the effects of foliar application of magnesium sulfate (MgSO_4_·7H_2_O) on the salt tolerance of rice under actual coastal saline-alkali soil conditions, bridging the gap between greenhouse/potted experiments and practical agricultural applications. The findings indicate that the response of rice to magnesium sulfate supplementation exhibits a threshold dependency under coastal saline-alkali conditions, with Mg²^+^ treatment achieving optimal results in both physiological and yield parameters. Under varying salinity levels of coastal saline-alkali soils, exogenous magnesium sulfate supplementation significantly enhanced rice yield by increasing chlorophyll content and Rubisco enzyme activity, thereby improving photosynthesis. Additionally, it minimized sodium (Na^+^) toxicity by regulating ion balance (increasing the ratios of Mg²^+^/Na^+^, K^+^/Na^+^, Ca²^+^/Na^+^, and P/Na^+^) and enhanced antioxidant enzyme activity to mitigate oxidative damage. The optimal exogenous magnesium sulfate treatment not only increased rice yield but also improved economic returns in saline-alkali environments, providing a sustainable and cost-effective strategy for rice cultivation in coastal saline-alkali soils. Even the study holds significant implications for guiding high-yield cultivation of rice in coastal saline-alkali areas and for the rational application of magnesium sulfate in the field, the lack of the control without salinity stress due to field conditions and specific molecular analysis on ion balance and antioxidant systems limited the systematicness and depth of the study. Thus, the molecular and omics approaches need to be integrated in the future work to elaborate on the mechanism by which magnesium regulates salt tolerance in rice.

## Data Availability

The original contributions presented in the study are included in the article/supplementary material. Further inquiries can be directed to the corresponding author.
